# California’s Public Safety Realignment Act and prisoner mortality

**DOI:** 10.1371/journal.pone.0284609

**Published:** 2023-04-28

**Authors:** Christopher L. Rowe, Alan Hubbard, Jennifer Ahern

**Affiliations:** 1 Division of Epidemiology, School of Public Health, University of California, Berkeley, Berkeley, California, United States of America; 2 Division of Biostatistics, School of Public Health, University of California, Berkeley, Berkeley, California, United States of America; University of Foggia: Universita degli Studi di Foggia, ITALY

## Abstract

In 2011, a historic Supreme Court decision mandated that the state of California substantially reduce its prison population to alleviate overcrowding, which was deemed so severe as to preclude the provision of adequate healthcare. To comply, California passed the Public Safety Realignment Act (Assembly Bill [AB] 109), representing the largest ever court-ordered reduction of a prison population in U.S. history. AB109 was successful in reducing the state prison population; however, although the policy was precipitated by inadequate healthcare in state prisons, no studies have examined its effects on prisoner health. As other states grapple with overcrowded prisons and look to California’s experience with this landmark policy, understanding how it may have impacted prisoner health is critical. We sought to evaluate the effects of AB109 on prison mortality and assess the extent to which policy-induced changes in the age distribution of prisoners may have contributed to these effects. To do so, we used prison mortality data from the Bureau of Justice Statistics and the California Deaths in Custody reporting program and prison population data from the National Corrections Reporting Program to examine changes in overall prison mortality, the age distribution of prisoners, and age-adjusted prison mortality in California relative to other states before and after the implementation of AB109. Following AB109, California prisons experienced an increase in overall mortality relative to other states that attenuated within three years. Over the same period, California experienced a greater upward shift in the age distribution of its prisoners relative to other states, suggesting that the state’s increase in overall mortality may have been driven by this change in age distribution. Indeed, when accounting for this differential change in age distribution, mortality among California prisoners exhibited a greater reduction relative to other states in the third year after implementation. As other states seek to reduce their prison populations to address overcrowding, assessments of California’s experience with AB109 should consider this potential improvement in age-adjusted mortality.

## Introduction

The United States (US) has both the largest prison population, with over two million current prisoners, and the highest incarceration rate in the world [1]. Although the country has recently started shifting away from more punitive and towards more rehabilitative forms of criminal justice and recent legislation has successfully decreased incarceration rates [2, 3], a substantial population remains incarcerated in prisons and jails in the US.

California has the nation’s second largest prison population. Following substantial population growth in the 1980’s and 1990’s, California’s prisons were characterized by extreme overcrowding during the 2000’s. Although California’s prisons were not unique in this regard, the circumstances surrounding its efforts to resolve the problem of overcrowding were. Two class action lawsuits filed on behalf California state prisoners alleged that inadequate mental (*Coleman v*. *Brown*, filed 1990) and physical (*Brown v*. *Plata*, filed 2001*)* healthcare stemming from overcrowded conditions violated prisoners’ constitutional rights. In connection with *Brown v*. *Plata*, federal courts established a receivership with full authority over the state’s prison healthcare system in 2006. Components of both lawsuits were ultimately consolidated before a single three-judge court, which in 2009 declared overcrowding to be the primary reason for healthcare deficiencies and mandated the state to reduce its prison population by nearly 50,000 individuals, corresponding to approximately a 30% reduction. This order was upheld by the U.S. Supreme Court in 2011 and California was required to meet the mandated reduction target within three years of the ruling [4]. To comply with the court’s decision, California passed the Public Safety Realignment Act (Assembly Bill [AB] 109) in October 2011.

AB109 sought to decrease the prison population by prospectively shifting the custodial responsibility of non-violent, non-serious, and non-sexual offenders from state prisons to county jails and probation departments, which was motivated by the idea that local agencies could do a better job of rehabilitating offenders [5, 6]. In addition, by limiting the funds provided to counties for these additional inmates, the policy incentivized more cost-effective alternatives to incarceration, such as the use of day reporting centers, shorter post-release community supervision, intensive probation, and home detention with GPS monitoring, with the aim of lowering the overall rate of incarceration across both prisons and jails [5, 6].

Empirical studies and policy analyses have examined the effects of AB109 on a variety of outcomes, including state prison and county jail populations [7–10], recidivism [7, 11–16], corrections spending [7], and crime [7, 8, 17–19]. Research suggests that AB109 significantly reduced the state prison population and extent of overcrowding, though not enough to reach the court-mandated target, while increasing the county jail population [7–9, 11]. The increase in the county jail population was smaller than the decrease in the state prison population, resulting in a lower rate of incarceration overall. However, one study found that AB109 may have exacerbated racial, ethnic, and gender disparities in incarceration in California’s prison system [10]. AB109 appears to have led to modest increases in recidivism statewide, but increases were smaller for counties that prioritized reentry programs [7, 11–15]. As of 2015, the policy appears to have had no effect on rates of violent crime, but may have increased rates of property crime, particularly auto theft [7, 8, 17–19].

Despite the fact that AB109 was precipitated by inadequate healthcare in California prisons, no independent studies have examined its effect on the health of prisoners. However, monthly reports tracking a wide variety of healthcare performance indicators did document improvements following the implementation of AB109 [20, 21]. Indeed, in 2013, Governor Jerry Brown of California declared that prison overcrowding no longer inhibited the delivery of timely and effective healthcare to prisoners.

AB109 was implemented amidst an evolving prison healthcare system in California, including documented improvements under the federal receivership and the opening of a new medical complex that was constructed in 2013 in response to decisions in both the *Plata* and *Coleman* cases [20]. As such, it is not possible to isolate the effects of AB109 from those due to other concurrent changes prompted by these lawsuits. However, given that overcrowding was identified as the primary reason for California’s inadequate prison healthcare [4], it is plausible that any changes in health outcomes over this period are at least partially attributable to the de-crowding effects of AB109. Furthermore, the changes initiated by AB109 were unprecedented in state-level criminal justice reform, representing the largest ever court-ordered reduction of a prison population in the U.S. Indeed, AB109 has been characterized as “the biggest criminal justice experiment ever conducted in America” [22]. At the end of 2020, 10 states exceeded at least one measure of prisoner capacity [23]. As these states continue to grapple with overcrowded prisons and look to California’s experience with AB109, it is critical that we understand how the policy may have impacted all relevant outcomes, including prisoner health.

Leveraging several complementary data sources, we aimed to evaluate the effects of AB109 on California state prisoner mortality. By shifting the responsibility of low-level offenders to county jails, AB109 impacted both the number of composition of prisoners in California state prisons. Because of these dynamics, we also sought to assess the extent to which effects of AB109 on prisoner mortality might be attributable contemporaneous changes in the age distribution of prisoners.

## Materials and methods

### Overview

To evaluate the effects of AB109 on California state prisoner mortality and assess the extent to which changes in the age distribution may have contributed to these effects, we conducted four complementary analyses. First, we evaluated the effects of AB109 on crude mortality (i.e., not accounting for temporal changes in age distribution) using the synthetic control method. Second, we examined how the age distribution of California state prisoners changed following AB109 and compared these changes to those occurring in other states over the same timeframe. Third, we compared trends in crude and age-standardized mortality among California state prisoners before and after AB109. Lastly, we compared pre- to post-AB109 changes in mortality among California state prisoners to those occurring in other states while accounting for differential changes in prisoner age distributions between states. This study was approved by the Committee for Protection of Human Subjects at the University of California, Berkeley (Protocol Number 2018-09-11405) and granted a waiver of informed consent because it only involved coded administrative data with no personal identifiers.

### Data sources

To comprehensively answer our research question, we would ideally leverage data on the universe of state prisoners in the US, including their ages and dates of incarceration, as well as if and when they died in prison. However, such comprehensive data were not obtainable. Instead, we leveraged three complementary data sources that were either publicly available or practically obtainable in order to conduct the four analyses outlined above.

First, we obtained the annual counts of deaths and crude mortality rates among state prisoners for all 50 states for years 2001–2015 from publicly available reports published by the Bureau of Justice Statistics (BJS) [24, 25].

Next, we obtained individual-level state prison inmate term records for California and several other states for years 2000–2015, including month and year of birth and prison term dates, from the National Corrections Reporting Program (NCRP) [26]. Annually, the NCRP collects offender-level administrative data on prison admissions and releases and yearend custody populations from participating jurisdictions. The number of states submitting data to NCRP has varied over time, with at least 38 states providing some amount of data since 2000. These data were available from the Inter-university Consortium for Political and Social Research under restricted conditions.

Lastly, we obtained de-identified individual-level data for all deaths among California state prisoners, including age of the decedent and date of death, from the California Deaths in Custody (DIC) reporting program [27]. The DIC Data includes all deaths that occur in law enforcement custody. To capture deaths among California state prisoners, we retained only deaths for which the California Department of Corrections and Rehabilitation had custody of the subject immediately preceding death. We excluded decedents held in out-of-state correctional facilities, those who died by execution, and those released on medical parole. These data were publicly available through California Department of Justice’s Open Justice program [28].

Collectively, these data sources provide us with or allow us to calculate crude annual mortality rates for California and comparison states (BJS Data), annual total mortality counts for California and comparison states (BJS Data), annual age-specific prisoner person-time for California and comparison states (NCRP Data), and annual age-specific mortality counts for California only (DIC Data). Supplemental Table 1 in S1 Appendix provides an overview as to which components of these data sources were used in each of our four analyses.

### Crude mortality

To evaluate the effect of AB109 on crude mortality, we used the synthetic control method with the annual crude mortality rates from the BJS as the outcome variable [29, 30]. Specifically, we considered California as the single treated unit, and included other states in the donor pool from which to construct the synthetic control. The synthetic control approach uses pre-policy covariates and outcome data to identify a weighted combination of control units whose weighted pre-policy covariates and outcomes best fit those of the treated unit. For our analysis, the pre-policy period included years 2001–2010 and the post-policy period included years 2012–2014. The year 2011 was excluded because AB109 was implemented in October 2011. Years after 2014 were excluded because California implemented Proposition 47, another major criminal justice law that reduced penalties for many low-level crimes, in November 2014. The effect of AB109 on prisoner mortality is estimated by comparing the observed California state prisoner mortality rate to that of the synthetic control unit in the post-policy period. Full methodological details of this analysis are provided in S1 Appendix.

### Changes in prisoner age distributions

In order to investigate the extent to which the effects of AB109 on crude mortality may be attributable to changes in California’s prisoner age distribution, we first sought to assess how the age distribution of California state prisoners changed following AB109 relative to other states over the same timeframe. If California experienced a change in age-distribution following AB109 that was extreme relative to concurrent changes in other states, this would support the plausibility that effects of AB109 on mortality could be attributable to a shift in the California’s prisoner age-distribution. To do this, we calculated the proportion of prisoner person-time corresponding to specific age groups (under 25, 25–34, 35–44, 45–54, 55–64, 65–74, over 74) for 2010 and each year 2012–2014 for California and each comparison state. Prisoner person-time was derived from the NCRP individual-level term records. Here, prisoner person-time for a given year refers to the total number of days incarcerated among all individuals incarcerated in that year; for example, if two prisoners were incarcerated in 2010, one for the entire year (i.e. 365 days) and one for only 30 days, these two prisoners would represent 365 + 30 = 395 days of prisoner person-time in 2010. For each state and year, calculating the proportion of prisoner person-time corresponding to specific age groups involved summing up the incarcerated person-time among prisoners falling within each age group and dividing by the total incarcerated person-time to obtain proportions. We then calculated the relative change in each age group proportion from 2010 to each of the three post-policy years for each state.

### Comparing crude and age-standardized mortality among California state prisoners

Next, we sought to investigate differences between the trend in crude California state prisoner mortality and the trend in California state prisoner mortality that accounts for changes in the age-distribution of prisoners over time and, particularly, following AB109. To do so, we compared the annual crude mortality rates among California state prisoners from 2008–2014 to annual age-standardized mortality rates over the same period, both calculated with death counts from the DIC data and person-time measures from the individual-level NCRP data. To calculate the annual age-standardize mortality rates, California’s annual age-specific mortality rates for each year from 2008–2014 were applied to the California state prisoner age-distribution from 2008, which preceded the implementation of AB109. This procedure is known as direct standardization. The age-standardized mortality rates for each year were thus calculated as:

$$\lambda_{CA,j}^{AS}=\frac{\sum_{k=1}^{K}\lambda_{CA,j,k}pt_{CA,2008,k}}{\sum_{k=1}^{K}pt_{CA,2008,k}}\;\mathrm{for}\;\mathrm{years}\;j=2008,2009,\ldots ,2014$$

Where λ^AS^_CA,*j*_ is the California age-standardized rate for year *j*; λ_*CA*,*j*,*k*_ is the California mortality rate for year *j* and age group *k*; and *pt*_*CA*,*2008*,*k*_ is the California incarcerated person-time for 2008 and age group *k*. The age-standardized mortality trend illustrates how crude mortality would have changed if the California state prisoner age-distribution were static from 2008 to 2014. We were only able to standardize California’s age-specific prisoner mortality rates in this way because we had access to annual age-specific mortality rates for California but not for any comparison states.

### Comparing age-standardized mortality between California and other states

Lastly, we sought to evaluate the effects of AB109 on mortality net of any effect due to resultant changes in the age distribution of California state prisoners and any secular trends approximated by the experience of other states. To do so, we compared California’s change in state prisoner mortality from pre- to post-policy years to that experienced by other states, while controlling for differential changes in age distribution between California and other states over the same period. Standard approaches to adjust for differential changes in age distribution between California and other states would require that annual age specific mortality rates among California and other states be compared directly or standardized to and summed over the same age distribution, such as California’s age distribution in a given year (i.e., direct standardization). However, as previously noted, the data available for this analysis precluded the ability to calculate age-specific rates for any state but California and thus we could not evaluate trends in age-adjusted mortality across states using the synthetic control method. We did, however, have access to the annual age-specific person-time from the NCRP data and the total number of deaths from the BJS data for comparison states.

To compare mortality trends between California and other states while accounting for differential changes in age distribution, this combination of data is amenable to an extension of the method of indirect age standardization, in which a set of “standard” age-specific mortality rates are standardized to and summed over a target age distribution to obtain the total number or rate of deaths expected in the target population had it experienced the “standard” age-specific mortality rates. We calculated a single measure for each control state and post-policy year that captured the excess (or deficit) change in mortality that would have occurred in the control state had it experienced California’s age-group specific mortality rates in the pre- and relevant post-policy year. Specifically, we used California’s annual age-specific mortality rates as the “standard” set of rates, which we standardized to and summed over the age distribution of each control state for each corresponding year. Thus, for each control state, we obtained four quantities that corresponded to the marginal mortality rate expected in each control state had it experienced California’s age-group specific mortality rates in 2010 and each year 2012–2014. Then, for each control state and post-policy year, we calculated the difference between the change in the expected marginal mortality rate from 2010 to a post-policy year and the change in the control state’s observed marginal mortality rate over the same period. Thus, the age-adjusted excess mortality was calculated as:

$$\begin{array}{c} EM_{i,j}=(\frac{\sum_{k=1}^{K}\lambda_{CA,j,k}pt_{i,j,k}}{\sum_{k=1}^{K}pt_{i,j,k}}-\frac{\sum_{k=1}^{K}\lambda_{CA,2010,k}pt_{i,2010,k}}{\sum_{i=1}^{K}pt_{i,2010,k}})-(CR_{i,j}-CR_{i,2010})\\ \mathrm{for}\;\mathrm{comparison}\;\mathrm{states}\;i=1,\ldots ,n\;\mathrm{and}\;\mathrm{years}\;j=2012,2013,2014 \end{array}$$

Where *EM*_i,j_ is the age-adjusted excess mortality for comparison state *i* and post-policy year *j*; λ_*CA*,*j*,*k*_ is the mortality rate for California in post-policy year *j* for age group *k*; *pt*_*i*,*j*,*k*_ is the incarcerated person-time for comparison state *i*, post-policy year *j*, and age group *k*; and *CR*_*i*,*j*_ is the crude mortality rate for comparison state *i* and post-policy year *j*. A positive value for this quantity suggests that the change in the marginal mortality rate associated with California’s pre- and post-policy age-specific mortality rates was greater than the change associated with the control state’s pre- and post-policy age-specific mortality rates, a negative value suggests the opposite, and a value of zero suggests that there was no difference between the two. This quantity can also be framed as a difference-in-differences estimate, for which California’s age-specific mortality rates applied to a control state’s age distribution represent the treated unit and the control states observed mortality rates represent the control unit.

For each post-policy year, we present the age-adjusted excess mortality rate estimates for each control state, the median value, and exact confidence intervals. Exact confidence intervals were obtained for the closest confidence levels above and below the 95% level via inversion of the one-sample sign test. The sign test is a non-parametric statistical test that makes no distributional assumptions about the underlying data distribution other than that data are drawn independently from a continuous distribution.

We also conducted a placebo test using 2008 as the pre-policy year and 2010 as the post-policy year and present the median and exact confidence intervals as described above. These years were selected so as to skip a single “implementation” year as in the main analysis and because 2010 is the last year prior to the actual implementation of AB109.

### Comparison states

A full summary of comparison states included in the crude and age adjusted mortality analyses and rationale for any exclusions are provided in S1 Appendix.

### Sensitivity analysis for differences in mortality reporting

As previously described, our comparison of changes in age-standardized mortality rates between California and other states leveraged multiple complementary datasets. There were small differences between the annual number of deaths among California state prisoners in the DIC and BJS data, with DIC data consistently reporting fewer deaths than the BJS data (Supplemental Table 3 in S1 Appendix). For the main analysis, we used the deaths present in the DIC data. To account for the possibility that DIC data are not comprehensive and that the BJS data represent the truth, we conducted a sensitivity analysis in which we added deaths to the DIC data such that the annual number of deaths matched the number in the BJS data for 2010 and each post-policy year 2012–2014. Over 1000 iterations, we randomly assigned the added deaths to different age groups (with equal probability), calculated the age-adjusted excess mortality rate estimates for each state and post-policy year, and calculated the median value for each post policy year. We then present the 2.5% and 97.5% quantiles of the distributions of the median values for each post-policy year.

### Bias analyses for concurrent policy changes

Two sources of potential bias in estimating the effects of AB109 on California state prisoner mortality are the nearly concurrent introduction of the medical parole program and the subsequent introduction of the elderly parole program in the California state prison system. The medical parole program began in January 2011, granted its first parole in June 2011, and was expanded in July 2014. This program initiated a parole hearing process that allowed grantees who are permanently medically incapacitated and who require 24-hour care to be placed in a licensed healthcare facility in the community. The elderly parole program began in October 2014 and initiated a parole hearing process by which prisoners over 60 years old who have served at least 25 years of continuous incarceration could be assessed for parole. An elderly parole hearing differs from a standard parole hearing in that the panel gives special attention to the prisoner’s age, physical condition, and long-term confinement when determining a prisoner’s suitability for parole.

These two programs may have implications for our analysis because they were introduced after the end of the pre-policy period and could have impacted the mortality rate among California state prisoners if those granted parole through the programs were at systematically higher or lower risk of death than those who remained incarcerated. If this were the case, any changes in mortality in the post-policy period could be at least partially due to the initiation of these parole programs.

We sought to assess the impact of these parole programs on the results of our comparison of age-standardized mortality rates between California and other states. It seems reasonable to assume that medical parolees would be at greater risk of death than other age-comparable prisoners, and thus their release from custody may have resulted in a reduction in age-specific prisoner mortality rates. However, two points are worth noting. First, medical parole differs from compassionate release, which involves a recall of sentence for California state prisoners who are terminally ill and are estimated to have less than six months to live. Although medical parolees are permanently medically incapacitated, they are not necessarily terminally ill. Second, there were never more than seven medical parole deaths during any post-policy year (Supplemental Table 4 in S1 Appendix), which represent a small fraction of the annual number of deaths among California state prisoners. Regarding the elderly parole program, we have no specific reason to expect elderly parolees to be more or less at risk of death than age-comparable prisoners who remained incarcerated.

To assess the sensitivity of our age-standardized analysis results to the introduction of the medical parole program, we calculated age-standardized excess mortality rates as described above but included deaths among California medical parolees and person time attributed among these deceased medical parolees in the calculation of the age-specific annual California mortality rates. Deaths among medical parolees were present in the DIC data and were excluded from our main analysis. In addition, we re-ran the sensitivity analysis described above to correct for differences in death counts between DIC and BJS data, but for each iteration we added medical parolee deaths after updating the annual DIC death counts to match those reported by BJS.

Because the medical parole program began after the end of the pre-policy period, including deaths among California medical parolees had no effect on the synthetic control fit for our crude mortality analysis. Their inclusion increased California’s observed mortality rate by <2% for each post-policy year and thus we do not present this sensitivity analysis for crude mortality.

To explore the potential impact of the elderly parole program on the California prison mortality rate, we compared the trend in the mortality rate among California state prisoners aged 60 and older before and after the start of the elderly parole program to the trend in rate among prisoners younger than 60. Specifically, we calculated mortality rates separately among prisoners above and below age 60 for each calendar quarter in 2013 and 2014. Because the variance of quarterly mortality rates among prisoners aged 60 and older were substantially larger than those among prisoners under 60 (60 and older variance = 100,463; under 60 variance = 530), we calculated the change in quarterly mortality rate since the rate in the first quarter (Q1) of 2013 standardized by the standard deviation of quarterly rates in 2013–2014 for each group. If the beginning of the elderly parole program led to a meaningful change in the mortality rate among prisoners aged 60 and older, we might expect to observe a standardized change in the mortality rate among this group in Q4 2014 that is not evident among prisoners under 60.

## Results

### Crude mortality

**Fig 1:**
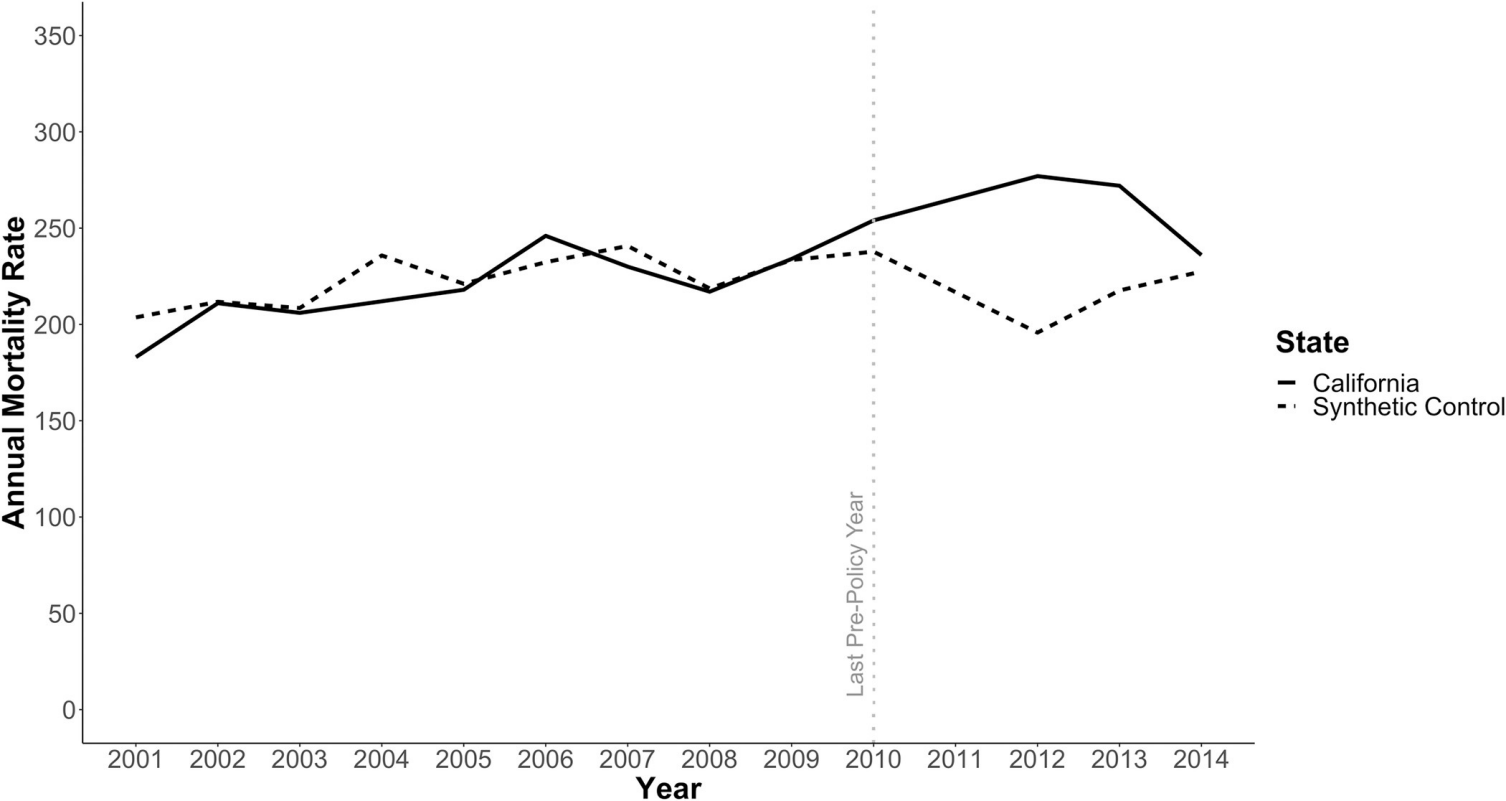
Annual prisoner mortality rate for California and synthetic control, 2001–2014.

**Fig 2:**
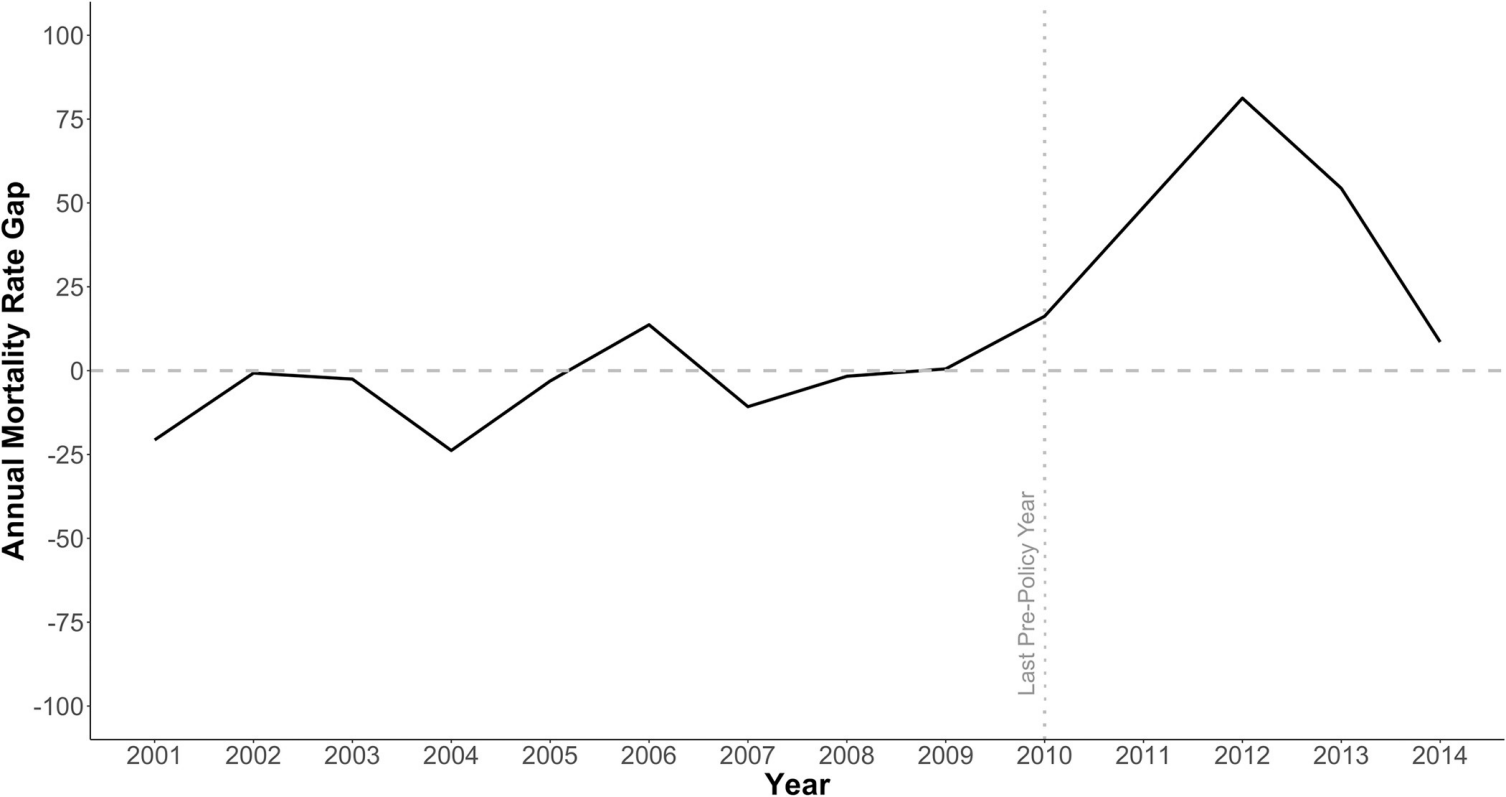
Annual prisoner mortality rate gap between California and synthetic control, 2001–2014.

The composition of the synthetic California is presented in Supplemental Table 5 in S1 Appendix, the crude mortality rates for California and the synthetic California are presented in Fig 1, and the difference between California’s annual mortality rate and those for the synthetic California are presented in Fig 2. Mortality among California state prisoners increased after the implementation of AB109 in 2011 relative to those of the synthetic California, but the two rates converged by 2014. These estimates suggest that, without accounting for age distribution changes, the mortality rate among California state prisoners was higher in 2012 and 2013 than would have been expected in the absence of AB109. Results of all permutation tests and robustness checks are provided in S1 Appendix.

It is worth noting that the 2012 crude mortality rate for California reported by the BJS is and used in this analysis likely to over-estimate the true mortality rate (in deaths per person-time). The BJS uses the year-end custody population as the denominator for calculating annual rates, and AB109 led to a large and rapid reduction in the prison population that did not stabilize until the end of 2012. Specifically, the yearend custody populations reported by BJS for 2011 and 2012 were 147,051 and 132,624, respectively. As a result of this reduction from yearend 2011 to yearend 2012, the yearend population will under-estimate the true incarcerated person-time more in 2012 than in other years.

### Changes in prisoner age distributions

**Fig 3:**
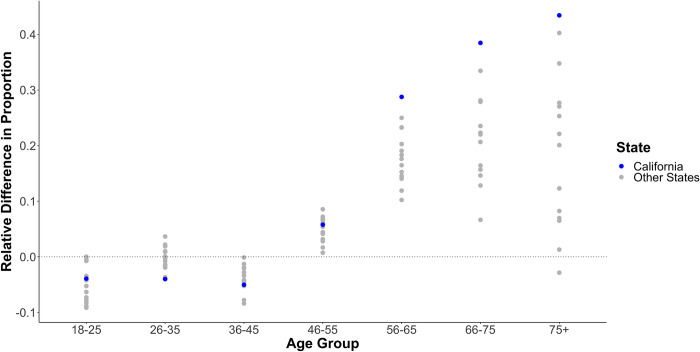
Relative change in proportion of person time by age group for 17 states with reliable National Corrections Reporting program data, 2010 to 2012.

After the implementation of AB109, the proportion of California state prisoners in older age groups increased and those in lower age groups decreased (Fig 3). From 2010 to 2012, California had relative increases in the proportion of prisoners in older age groups that exceeded those of most control states with reliable person-time data (53% of control states for prisoners aged 45–54 and 100% of control states for prisoners aged 55–64, 65–74, and over 74). Similarly, California had relative decreases in the proportion of prisoners for some younger age groups that exceeded those of most control states (80% of control states for prisoners aged 35–44; 100% of control states for prisoners aged 25–34; 33% of control states for prisoners aged 18–24). Similar patterns were sustained through 2013 and 2014 (Supplemental Figs 12–13 in S1 Appendix).

### Comparing crude and age-standardized mortality among California state prisoners

**Fig 4:**
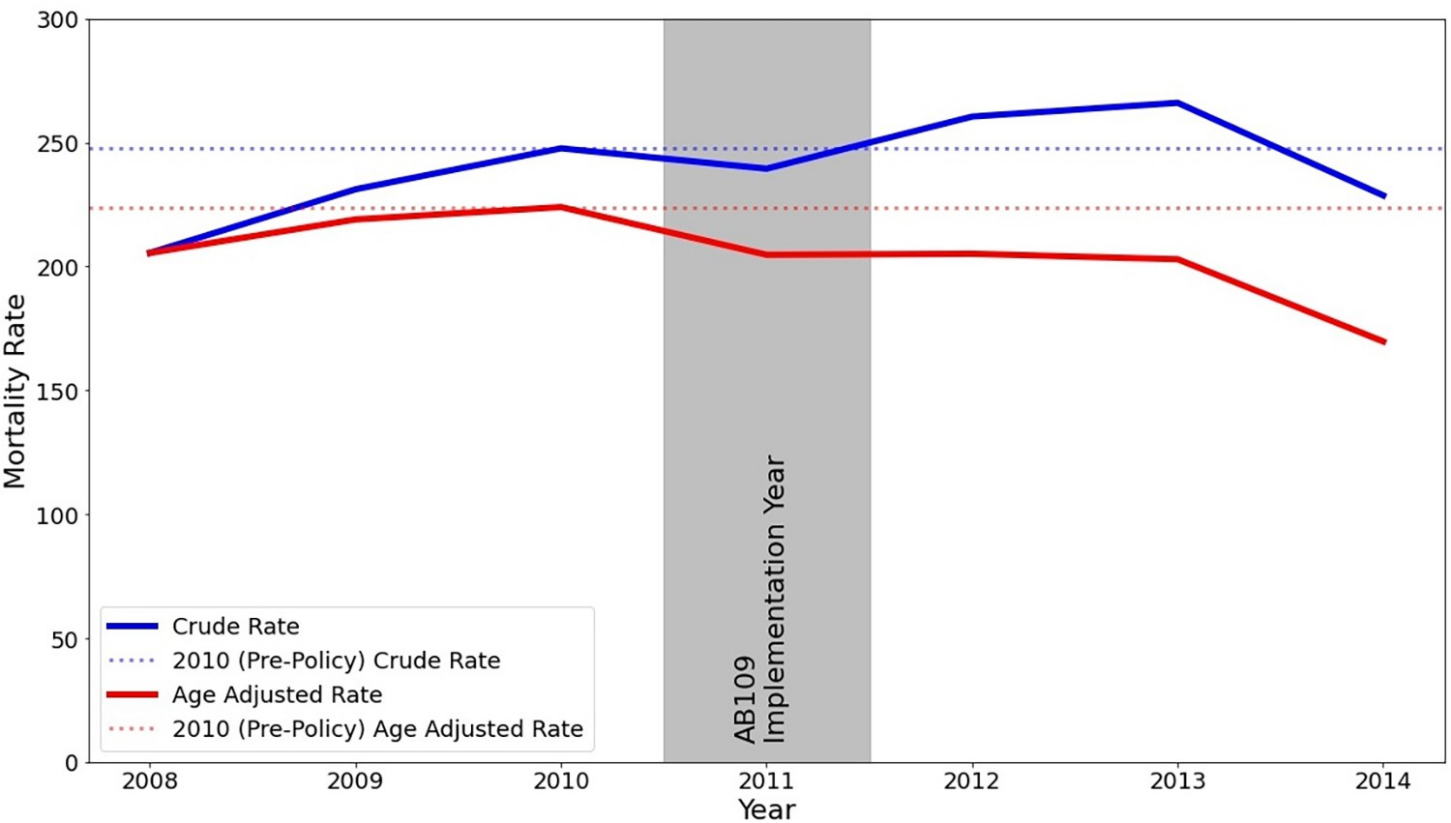
Crude and age-adjusted California prisoner mortality rates, 2008–2014.

Both the crude and age-standardized mortality rates among California state prisoners from the period 2008–2014 are presented in Fig 4. The increase that was observed for crude mortality rates following the implementation of AB109 is absent from the age-standardized mortality rates. Whereas the crude mortality rate increased from 247.7 deaths per 100,000 person-years in 2010 to 260.5 in 2012 and 266.0 in 2013, the age-standardized mortality rate decreased from 223.9 in 2010 to 205.1 in 2012 and 202.9 in 2013. We also present the age-specific mortality counts and rates among California state prisoners for each year from 2008 to 2014 in Supplemental Tables 6–7 in S1 Appendix. Age-specific mortality rates declined for all but two age groups (under 25 and 45–54) from 2010 to 2012 and declined for all but one age group (under 25) from 2010 to 2013 and 2014.

### Comparing age-standardized mortality between California and other states

**Fig 5:**
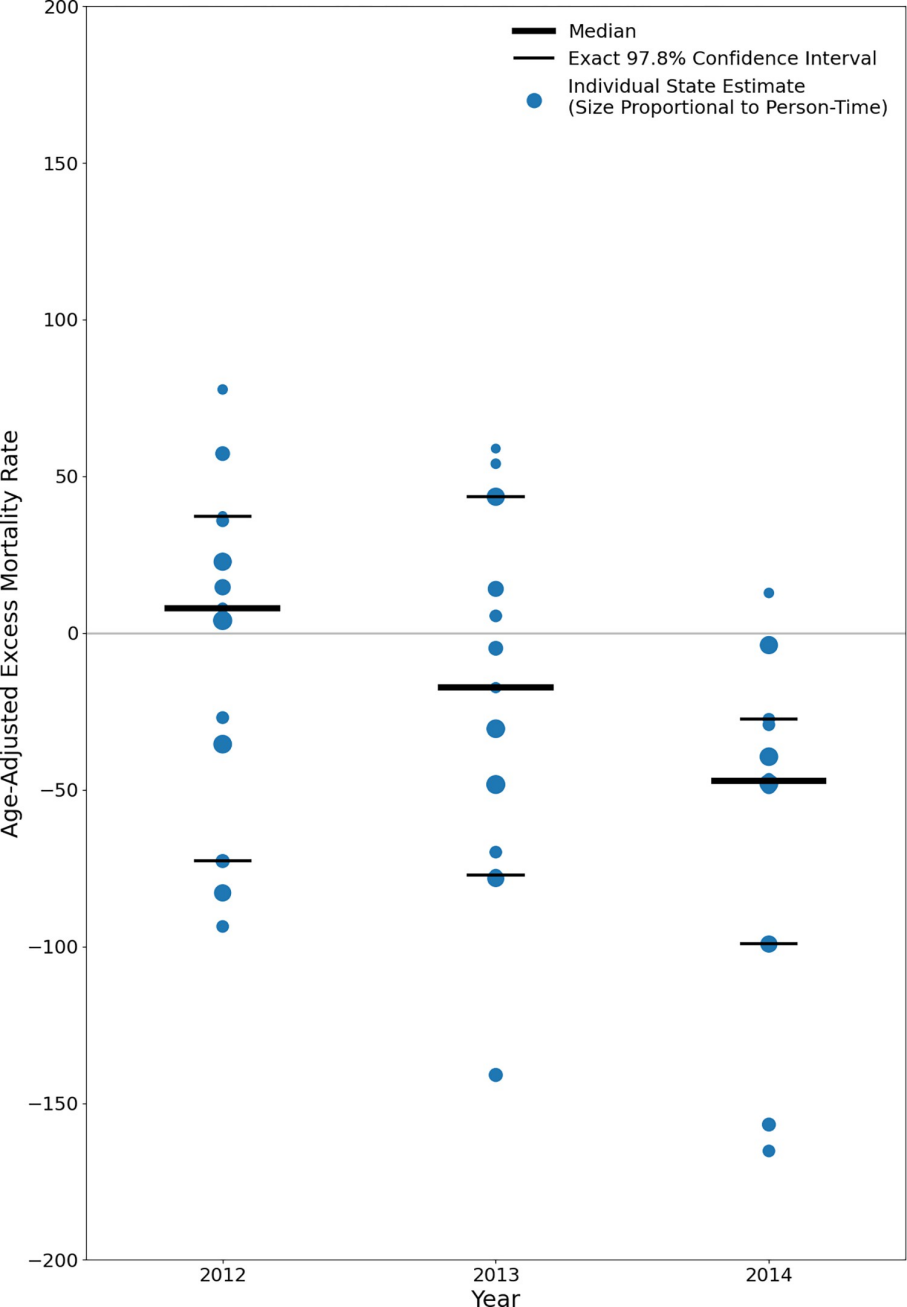
Age-adjusted excess mortality rates, 13 states, 2012–2014.

The age-standardized excess mortality rate estimates for each state and post-policy year are presented in Fig 5 and Supplemental Table 8 in S1 Appendix. Among the 13 control states included in the analysis, the median excess mortality rates were 7.9 (97.8% exact confidence interval: -72.8, 37.3; 90.8% exact confidence interval: -35.5, 35.8) in 2012, -17.4 (-77.2, 43.5; -69.9, 14.1) in 2013, and -47.1 (-99.2, -27.5; -49.0, -29.3) in 2014. These results suggest that if each control state’s prison population had experienced California’s age-specific mortality rates from 2010 to 2014, they would have tended to have greater decreases in mortality relative to what they actually experienced. By 2014, the median state would have experienced a nearly 50 fewer deaths per 100,000 prisoners, representing a nearly 20% reduction from the median mortality rate of 259 per 100,000 among the 13 control states in that year.

When applied to the pre-policy period (2008 to 2010), the median excess mortality rate was 36.4 (-3.2, 108.6; 20.2, 84.4). This result suggests that if each control state’s prison population had experienced California’s age-specific mortality rates from 2008 to 2010, they would have tended to have greater increases in mortality relative to what they actually experienced.

There was no evidence that the changes in crude mortality among states included in the analysis arise from a different distribution than excluded states from 2010 to 2012 (*p* = 0.402), 2013 (*p* = 0.806*)*, or 2014 (*p* = 0.943*)*, as assessed using Kolmogorov-Smirnov tests.

### Sensitivity analysis for differences in mortality reporting

The results of our sensitivity analysis in which we randomly corrected for differences between the annual numbers of California state prisoner deaths included in the DIC data and those reported by BJS were consistent with our main age-standardized analysis results comparing changes in California to those in other states and are presented in Supplemental Table 9 in S1 Appendix.

### Bias analyses for concurrent policy changes

The results of our sensitivity analysis that incorporated medical parole deaths were also similar to our main age-standardized analysis results comparing changes in California to those in other states (Supplemental Table 10 in S1 Appendix). As with our main analysis, randomly correcting for differences between DIC and BJS data had little effect on these estimates (Supplemental Table 11 in S1 Appendix).

In regards to the elderly parole program, the standardized changes over time in quarterly mortality rates among prisoners above and below 60 years of age were very similar throughout 2013 and 2014, with the exception of a spike in deaths among prisoners 60 and older in Q1 2014 (Supplemental Figure 14 in S1 Appendix). Under the assumption that the relationship between the mortality trends among those above and below 60 years of age would have continued through Q4 2014 in the absence of the elderly parole program, there is no indication that the elderly parole program impacted mortality rates among prisoners aged 60 and over.

## Discussion

Following the implementation of AB109, California prisons experienced an increase in crude mortality that attenuated by 2014 and appears to be attributable to a concurrent shift in the age distribution of California state prisoners. When accounting for this change in the age distribution, California’s change in mortality rate appeared consistent with those of other states in the first two post-policy years but exhibited a greater reduction relative to other states in the third year after implementation. This is the first study evaluating California state prisoner mortality following the implementation of California’s historic criminal justice initiative, and our analysis suggests improved mortality outcomes three years after implementation.

The results of our synthetic control analysis suggest that the crude mortality among California state prisoners increased relative to what would have occurred in the absence of AB109, with the largest increase occurring in 2012. This finding is supported by our permutation test when restricting to control states with good pre-policy fits and our robustness checks. However, as previously noted, the crude mortality among California state prisoners that is reported by BJS and used in this analysis over-estimates the true mortality rate in 2012, thus the magnitude of the effect in this year is likely exaggerated. Importantly, our age-focused analyses suggest that this increase is attributable to a substantial shift in the age distribution of California state prisoners as opposed to other mechanisms elevating prisoners’ mortality risk. We explored this possibility in three ways.

First, we found that California experienced a shift in age distribution among prisoners that was more extreme than most comparison states, particularly in regards to increasing proportions of older prisoners, following the implementation of AB109. AB109 provisions were implemented prospectively such that that new low-level offenders would be incarcerated in county jails instead of state prisons but existing state prisoners would not be transferred to county jails or granted early release. Although both admissions and releases declined after AB109, the reduction in California’s prison population was driven by a decline in admissions that exceeded the corresponding decline in releases. The rapid change in the age distribution of California’s prisoner population following AB109 aligns with the fact that that older prisoners are more likely to be serving longer sentences [31], such that prisoners released in a given year are disproportionately younger than the overall prisoner population. Prior to AB109, these releases would be approximately offset by new admissions, but new admissions were reduced under AB109, a dynamic that resulted in a smaller and older prisoner population. It is well established that mortality risk increases with age both in and out of prisons [24, 32], thus it is plausible that the age-shift precipitated by AB109 could increase the crude mortality rates.

Second, we found that the California state prisoner age-specific mortality rates actually declined for nearly all age groups following the implementation of AB109. This is also reflected in our finding that while the crude mortality rate increased from 2010 to 2012 and subsequently declined through 2014, the age-standardized rate declined over the entire period.

Lastly, in our comparison of changes in age-standardized mortality rates between California and other states, we found no evidence that changes in California age-specific mortality rates were systematically different from those of 13 comparison states from 2010 to 2012 or 2013, but we did find that California’s age-specific rates decreased more than those of all but one comparison state in 2014. Furthermore, we presented evidence that if each comparison state had experienced California’s age-specific mortality rates before and after AB109, the median state would have experienced nearly 50 fewer deaths per 100,000 prisoners compared to what was actually observed, or approximately 20% relative to the median 2014 mortality rate among comparison states.

Taken together, our findings suggest that crude mortality increased among California state prisoners following AB109 due to an evident change the age distribution and corresponding risk profile of California’s incarcerated population, but that mortality within age strata declined both relative to the year before implementation and relative to contemporaneous changes in other states. Understanding the overall (i.e. crude) mortality risk of the incarcerated population is critical for surveillance and healthcare planning; however, to understand how the de-population of California’s state prisons under AB109 may have impacted the health of individual prisoners, it is essential to account for the concurrent change in age distribution and the corresponding mortality risk.

The discrepant findings between our crude and age-adjusted analyses highlight an important methodological consideration when studying trends or impacts of policies on population measures. Specifically, researchers must take special care when applying methods and interpreting results when the internal composition of an aggregate population being studied may change in a manner that impacts the outcome of intertest. In the present scenario, a careless interpretation of the results of our synthetic control analysis might conclude that AB109 increased the mortality risk among California state prisoners, which our age-adjusted analyses suggest is not the case. Importantly, any approach effectively accounting for such dynamics requires data at a level more granular than crude population measures (e.g., individual-level data or measures stratified by relevant covariates), which might not always be available.

It is important to place our findings within the broader context of the California prison healthcare system leading up to and following the implementation of AB109. As noted in the Introduction, California’s prison healthcare system has a unique and complex history dating back decades from the implementation in AB109. Most notably, from 2006 through the end of the study period, California’s healthcare system was under full authority of a federal receiver specifically tasked with improving the quality of care [20]. From 2008 to 2010, both the crude and age-standardized mortality rates among California state prisoners increased; in fact, when calculating age-standardized excess mortality rates for this period, California appeared to have a greater increase in age-specific mortality rates than comparison states. Despite these increases in the early years of the receivership, a 2015 report by the receiver summarized substantial improvements to the structure, processes, and outcomes related to medical care in California prisons since 2008, though it also noted extensive variability in quality of care at the institutional level [20]. Also, from 2006 to 2014, a comprehensive death review process documented a reduction in the rate of medically preventable deaths [33]. In addition to these changes that spanned the pre- and post-AB109 periods, the state opened the California Health Care Facility (CHCF) in July 2013. The CHCF is a 54-building and nearly 3000-prisoner capacity medical complex for prisoners with long-term medical or acute mental-health needs. It is thus clear that the de-population of California prisons under AB109 occurred in tandem with dedicated and seemingly effective efforts to improve the quality of medical care provided to prisoners. Indeed, although the original court decision and subsequent Supreme Court opinion for *Coleman/Plata vs*. *Brown* declared overcrowding as the primary reason for healthcare deficiencies, they also made clear that resolving overcrowding alone would not be sufficient to improve the quality of care for prisoners.

The changes that spanned or occurred during the post-policy period offer potential explanations for why we only observed the reduction in age-adjusted prisoner mortality in 2014, the third year after the implementation of AB109. Although the CHCF opened in July 2013, the receiver halted intake in January 2014 citing inadequate staffing and supply chain issues that precluded effective provision of healthcare at the facility [34]. Following a series of improvements, intake at CHCF resumed in July 2014 [35]. Thus, after a challenging first 6–12 months in operation, CHCF may have impacted system-wide mortality rates by admitting and providing care for prisoners with the most complex and severe medical needs in 2014. Indeed, CHCF’s average end-of-month population was 366 across all 12 months in 2013 and 1,620 in 2014 [36]. In addition, the state’s death review process determined that the rate of medically preventable deaths was relatively stable from 2009 to 2013 but decreased by one-third in 2014, which aligns with our findings [33]. On the other hand, the average number of identified lapses in care per prisoner death showed no meaningful trend prior to 2010 but decreased from 1.1 in 2010 to 0.8 in 2012–2013 and to 0.5 in 2014 [33], which aligns with the timing of AB109 implementation. It is worth noting that death is the most severe health outcome and that improvements in medical care are likely to impact intermediate health outcomes before ultimately impacting mortality. This is particularly true in the context of chronic conditions such as diabetes or hypertension, which require ongoing care and management and are more prevalent among incarcerated populations [37]. In addition, the decline in California’s prison population resulting from AB109 mostly occurred in the first year after implementation, stabilizing around 133,000 inmates by September 2012 [38]. Accordingly, it seems plausible that de-population under AB109, not fully realized until late 2012, could have facilitated subsequent improvements in the quality of prisoner care that would correspond to the observed lag between the implementation of AB109 and changes in mortality.

Our study is novel in that it evaluates the impacts of decarceration on individuals who remain incarcerated and that it focuses on a health outcome. Prior work has noted the potential for decarceration efforts to improve the health and well-being of prisoners by reducing overcrowding and decreasing prisoner-to-staff ratios [39]; indeed, AB109 was predicated on this idea [4]. However, as illustrated by other research evaluating AB109 [7, 8, 11–19], outcomes related to public safety and recidivism tend to receive more attention among efforts to understand the impacts of decarceration. Our findings that AB109 may have decreased the risk of mortality among California state prisoners provides an important complement to this other work, and allows for a more comprehensive view of the impacts of AB109 and of decarceration more broadly.

The present study has several limitations. First, our synthetic control analysis and our age-standardized excess mortality rate measures both rely on the assumption that the mortality experience of other states can be used to approximate what would have occurred in California in the absence of AB109, which is untestable. Second, we are unable to disentangle the mortality effects of de-population under AB109 and concurrent improvements in the quality of medical care in California’s prisons that were prompted by the same lawsuits as AB109; however, given that these were intended as concurrent and complementary strategies for improving prisoner healthcare, we are not certain that it would be meaningful to do so. Third, only 29 and 13 control states were included in the crude and age-adjusted analyses, respectively; however, the majority of states were excluded due to small population sizes and numbers of prisoner deaths or having integrated prison and jail systems, which may limit their comparability to California, which has the nation’s second largest state prison system. In addition, among states included in the crude mortality analysis, there was no difference in the distributions of crude mortality trends between those that were included and excluded from the age-adjusted analysis. Fourth, our age-adjusted analysis combines data from multiple sources, which involved population data of variable quality in NCRP data and discordant counts of annual deaths among California state prisoners in the BJS and DIC data. We sought to address the variable quality in the NCRP data by validating year-end custody counts against NPS data and only retaining states that exhibited reasonable quality over the entire study period. We sought to address the discordant death counts in our stochastic sensitivity analysis, which had trivial effects on our estimates and no impact on our conclusions. It is important to note that broader availability of reliable data on US prison populations, including health-related measures, would mitigate the majority of these limitations and improve the ability of researchers to rigorously evaluate the impacts of changes to the nation’s prison systems. Indeed, a study surveyed 36 prominent publicly available health datasets and found the none could be used to assess the health of incarcerated individuals, highlighting the dearth of these data [40].

Despite these limitations, we leveraged multiple data sources to examine mortality among California state prisoners following a historic and unprecedented de-populating of a state prison system. Although the crude mortality rate increased initially, our findings suggest this was due to an expected shift in age distribution of California state prisoners. When accounting for this shift in age distribution, we found that mortality decreased more in California relative to comparison states three years after the implementation of AB109. As other states seek to reduce their prison populations to address overcrowding and corresponding healthcare deficiencies, any assessment of California’s experience with AB109 should consider this potential benefit.

## Supporting information

S1 Appendix Supplementary methods, results, tables, and figures. (DOCX)

S1 Dataset (ZIP)

## References

[1] R W. World Prison Population List, 12th Edition. London, UK: Institute for Criminal Policy Research (ICPR) at Birkbeck, University of London; 2018.

[2] Bronson J, Carson EA. Prisoners in 2017. Bureau of Justice Statistics, Office of Justice Programs, U.S. Department of Justice; 2019.

[3] Schrantz D, DeBor S, Mauer M. Decarceration strategies: How 5 states achieved substantial prison population reductions. Washington, D.C.: The Sentencing Project; 2018.

[4] Brown v. Plata. 2011.

[5] Misczynski D. Corrections Realignment: One Year Later. San Francisco, CA: Public Policy Institute of California; 2012.

[6] Lofstrom M, Bird M, Martin B. California’s Historic Corrections Reforms. San Francisco, CA: Public Policy Institute of California; 2016.

[7] Lofstrom M, Martin B. Public Safety Realignment: Impacts So Far. San Francisco, CA: Public Policy Instute of California; 2015.

[8] Lofstrom M, Raphael S. Realignment, Incarceration, and Crime Trends in California. San Francisco, CA: Public Policy Institute of California; 2015.

[9] Lofstrom M, Raphael S. Impact of Realignment on County Jail Populations. San Francisco, CA: Public Policy Institute of California; 2013.

[10] Gottlieb A, Charles P, McLeod B, Kjellstrand J, Bonsu J. Were California’s Decarceration Efforts Smart? A Quasi-Experimental Examination of Racial, Ethnic, and Gender Disparities. Criminal Justice and Behavior. 2020; 009385482092338. doi: 10.1177/0093854820923384

[11] Wootton A. AB 109 and its impact on prison overcrowding and recidivism: A policy analysis. Themis: Research Journal of Justice Studies and Forensic Science. 2016;4.

[12] Bird M, Grattet R, Nguyen V. Realignment and Recidivism in California. San Francisco, CA: Public Policy Institute of California; 2017.

[13] Bird M, Grattet R. Do Local Realignment Policies Affect Recidivism in California? San Francisco, CA: Public Policy Institute of California; 2014.

[14] Lofstrom M, Raphael S, Grattet R. Is Public Safety Realignment Reducing Recidivism in California? San Francisco, CA: Public Policy Institute of California; 2014.

[15] Bird M, Grattet R. Realignment and recidivism. The ANNALS of the American Academy of Political and Social Science. 2016;664: 176–195.

[16] Bird M, Grattet R. Policy change and recidivism: the effects of California realignment and local implementation strategies on rearrest and reconviction. Criminal Justice Policy Review. 2015;28: 601–623.

[17] Lofstrom M, Raphael S. Public Safety Realignment and Crime Rates in California. San Francisco, CA: Public Policy Institute of California; 2013.

[18] Sundt J, Salisbury EJ, Harmon MG. Is downsizing prisons dangerous? The effect of California’s realignment act on public safety. Criminology and Public Policy. 2016;15: 315–341.

[19] Lofstrom M, Raphael S. Incarceration and crime: evidence from California’s public safety realignment reform. The ANNALS of the American Academy of Political and Social Science. 2016;664: 196–220.

[20] Kelso J.C. Special Report: Improvements in the Quality of California’s Prison Medical Care System. 2015 Mar. Available: https://cchcs.ca.gov/wp-content/uploads/sites/60/2017/08/Kelso-Special-Report-Filed-031015.pdf

[21] California Correctional Health Care Services. Health Care Services Dashboard. [cited 31 Aug 2020]. Available: https://cchcs.ca.gov/reports/#dashboard

[22] Petersilia J. Looking back to see the future of prison downsizing in America. Keynote Address presented at: National Institute of Justice (NIJ) Conference; 2012 Jun. Available: https://www.ojp.gov/library/publications/looking-back-see-future-prison-downsizing-america-keynote-address-2012-nij

[23] Carson EA. Prisoners in 2020—Statistical Tables. Bureau of Justice Statistics, Office of Justice Programs, U.S. Department of Justice; 2021.

[24] Carson EA, Cowhig MP. Mortality in State and Federal Prisons, 2001–2016—Statistical Tables. United States Department of Justice, Office of Justice Programs, Bureau of Justice Statistics; 2020 Feb. Report No.: NCJ 251920.

[25] Noonan ME. Mortality in State Prisons, 2001–2014—Statistical Tables. United States Department of Justice, Office of Justice Programs, Bureau of Justice Statistics; 2016 Dec. Report No.: NCJ 250150.

[26] United States Department Of Justice. Office Of Justice Programs. Bureau Of Justice Statistics. National Corrections Reporting Program, 2000–2015: Version 1. ICPSR—Interuniversity Consortium for Political and Social Research; 2017. doi: 10.3886/ICPSR36746.V1

[27] State of California, Department of Justice, Criminal Justice Statistics Center. Deaths in Custody Data, 2005–2018. 2019.

[28] California Department of Justice. Open Justice. 2020 [cited 30 Sep 2020]. Available: https://openjustice.doj.ca.gov/

[29] Abadie A, Diamond A, Hainmueller J. Synthetic control methods for comparative case studies: Estimating the effect of California’s tobacco control program. Journal of the American Statistical Association. 2010;105: 493–505.

[30] Abadie A, Diamond A, Hainmueller J. Comparative Politics and the Synthetic Control Method. American Journal of Political Science. 2015;59: 495–510. doi: 10.1111/ajps.12116

[31] Urban Institute. A Matter of Time: The Causes and Consequences of Rising Time Served in America’s Prisons. Washington, D.C.; 2017 Jul. Available: https://apps.urban.org/features/long-prison-terms/intro.html

[32] Xu J, Murphy S, Kochanke K, Arias E. Mortality in the United States, 2018. Hyattsville, MD: National Center for Health Statistics; 2020.

[33] Imai K. Analysis of 2014 Inmate Death Reviews in the California Correctional Healthcare System. 2015 Jul. Available: https://cchcs.ca.gov/wp-content/uploads/sites/60/2017/08/OTRES_DeathReviewAnalysisYear2014_20150730.pdf

[34] California Correctional Health Care Receivership. Achieving a Constitutional Level of Medical Care in California’s Prisons: Twenty-fifth Tri-Annual Report of the Federal Receiver’s Turnaround Plan of Action For September 1—December, 2013. 2014 Feb.

[35] California Correctional Health Care Receivership. Achieving a Constitutional Level of Medical Care in California’s Prisons: Twenty-seventh Tri-Annual Report of the Federal Receiver’s Turnaround Plan of Action For May 1—August 31, 2014. 2014 Oct.

[36] California Department of Corrections and Rehabilitation. Monthly Reports of Population, July 2013—December 2014.

[37] Maruschak L, Berzofsky M, Unangst J. Medical Problems of State and Federal Prisoners and Jail Inmates, 2011–2012. U.S. Department of Justice. Office of Justice Programs. Bureau of Justice Statistics.; 2015.

[38] California Department of Corrections and Rehabilitation. Monthly Reports of Population, September 2011— September 2012.

[39] Garland B, Hogan N, Wodahl E, Hass A, Stohr MK, Lambert E. Decarceration and its possible effects on inmates, staff, and communities. Punishment & Society. 2014;16: 448–473. doi: 10.1177/1462474514539535

[40] Ahalt C, Binswanger IA, Steinman M, Tulsky J, Williams BA. Confined to ignorance: the absence of prisoner information from nationally representative health data sets. J Gen Intern Med. 2012;27: 160–166. doi: 10.1007/s11606-011-1858-7 21922160 PMC3270223

