# Supplementary material for: California’s Public Safety Realignment Act and prisoner mortality
Source: PLoS One. 2023 Apr 28;18(4):e0284609. doi: 10.1371/journal.pone.0284609 (PMC10146482; doi:10.1371/journal.pone.0284609)
Supplement: S1 Appendix — (DOCX) [file pone.0284609.s001.docx]

**S1 Appendix: Supplementary methods, results, tables, and figures**

**METHODS**

*Crude Mortality*

In our implementation of the synthetic control method, we included all annual values of the pre-policy prison mortality rates and no other covariates in the construction of the synthetic control unit. We considered several covariates but obtained a substantially improved pre-policy fit with regard to root mean squared prediction error when including only pre-policy outcomes. Specifically, we considered the following state-level covariates: age-adjusted all-cause mortality rate among the general population, incarceration rate, proportion of male prisoners, proportion of prisoners over 55, and prison population as a percentage of the lowest reported measure of prison facility capacity.

General population mortality rates were obtained from the Centers for Disease Control and Prevention’s Wide-Ranging Online Data for Epidemiologic Research (CDC WONDER) database;^1^ incarceration rates, proportions of male prisoners, and capacity measures were obtained from annual BJS Prisoners Series reports;^2^ and the proportions of prisoners over 55 were obtained from NCRP data that was processed and published by the Prison Policy Initiative.^3^ Each prison-related measure referred to the prison population at year end.

The synthetic control approach precludes traditional approaches to statistical inference; however, it is standard to conduct inference using a permutation test to compare the estimated treatment effect for the state that implemented the policy under study to estimates for states where policy was not implemented.^4,5^ Specifically, we applied the synthetic control estimator to every state in the control donor pool for the same time period assessed for the main analysis and compared the estimated treatment effect to these placebo effects in two ways: (1) comparing the post-policy mean squared prediction error (i.e., the sum of squared differences between the observed and synthetic mortality rates over the post-policy years) (MSPE) for California’s synthetic control to those of each of the control states, while restricting to subsets of states with good pre-policy fits (i.e., within 5x and 2x of the pre-policy MSPE for California’s synthetic control); and (2) comparing the ratio of the post- to pre-policy MSPE for California’s synthetic control to those of each of the control states. This latter measure precludes choosing an arbitrary cutoff for excluding control states with poor pre-policy fits by standardizing post-policy effect sizes by the quality of the pre-policy fit. The permutation test provides a distribution of placebo effects for each of these measures to which we can compare the values for California. We generated plots to visualize the results and calculated the proportion of control states that had post-policy MSPEs and ratios of post- to pre-policy MSPEs that were less extreme than those of California. Each of these inferential approaches have been described in greater detail elsewhere.^4,5^

We also conducted two separate robustness checks. First, we conducted an “in-time” placebo test, in which we applied the synthetic control method to California but considered 2001-2006 as the pre-policy period, 2007 as the excluded implementation year, and 2008–2010 as the post-policy period and also conducted a permutation test as described above. These years were selected because they allowed for the greatest number of pre-policy years while maintaining three post-policy years that occurred prior to the actual implementation of AB109. Because AB109 was not implemented until 2011, we should expect the mortality rates of the synthetic control unit to mirror those observed in California from 2008-2010. Second, for each control state that contributed to the synthetic control unit in the main analysis, we constructed a new synthetic control for California while excluding that state from the donor pool. We then compare the outcome trajectories of these leave-one-out synthetic control units to those of the original synthetic control unit that included the entire donor pool. These comparisons assess the robustness of our main estimates to the exclusion of any single state.

*Comparison States*

For both the crude and age adjusted mortality analyses, we excluded several control states. Specifically, we excluded states that had 10 or fewer deaths during any year under study. The BJS considers 10 or fewer deaths an insufficient number to provide a reliable rate.^6,7^ Florida was excluded because the state privatized its prison healthcare system in 2011, which an independent audit found to correspond with decreased quality of care.^8^ Alaska, Connecticut, Delaware, Hawaii, Rhode Island, and Vermont were excluded because prisons and jails form one integrated system in these states and mortality data were only available among the combined prison and jail populations.^6,7^

For the age-adjusted analysis, we excluded additional states for which the person time measures calculated using the NCRP data were determined to be inaccurate. Through the National Prisoner Statistics (NPS) Program, the BJS collects and reports the yearend custody population for each state prison system (i.e., the number of prisoners in physical custody on December 31 of each year).^9,10^ Separately, we used the individual-level term records from the NCRP to calculate the yearend custody population for each state and year for 2007-2014. We excluded states for which the yearend custody population calculated using NCRP data differed by more than 10% from the number reported by BJS during any year. A 10% accuracy threshold between NCRP and NPS data has been used in prior analyses using NCRP data.^11,12^

The inclusion status and reasons for exclusion for all 50 states for both the crude and age-adjusted mortality analyses are presented in Supplemental Table 2.

Because we do not have access to age-specific mortality rates for any control states, we cannot examine whether there were systematic differences in these rates between the states that were included and excluded from the age-adjusted analysis. However, we investigated whether the changes in crude mortality rates from 2010 to each post policy year from the BJS data are different by inclusion status for the age-adjusted analysis. Specifically, we assess whether the changes in each group (included vs. excluded) are likely to arise from the same underlying distribution using Kolmogorov-Smirnov tests for each post policy year. For this assessment, we only consider states that were included in the crude mortality analysis.

**RESULTS**

*Crude Mortality*

Plots for the permutation test are presented in Supplemental Figures 1-4. Specifically, the plots of the gaps between each control state’s mortality rate and that of their corresponding synthetic control are presented for all control states (Supplemental Figure 1) and those within 5x (Supplemental Figure 2) and 2x (Supplemental Figure 3) of California’s pre-policy MSPE. Nine (31.0%) of all 29 control states, five (26.3%) of 19 states within 5x of California’s pre-policy MSPE, and zero (0.0%) of 7 states within 2x of California’s pre-policy MSPE had a post-policy MSPE greater than that of California. The plot of the ratios of post- to pre- policy MSPE for California and all control states is also presented (Supplemental Figure 4). Three (10.3%) of 29 control states had ratios of post- to pre-policy MSPE greater than that of California.

A sizeable proportion of control states had greater post-policy MSPE values than California, yet few of these states had particularly good pre-policy fits. When restricting to seven states with good pre-policy fits, California’s estimated effect was more extreme than those of all placebo states. Similarly, California’s ratio of post- to pre-policy MSPE, which standardizes effect size by the quality of the pre-policy fit, exceeded those of 90% of placebo states. Thus, when accounting for pre-policy fit, the estimated effect of AB109 on mortality is relatively large compared to the distribution of placebo effects. These results provide modest, but not conclusive, evidence that the estimated effect in Figure 1 is due to AB109.

For the “in-time” placebo test treating 2007 as the policy implementation year, the crude mortality rates for California and the synthetic control and the difference between California’s annual mortality rate and those for the synthetic California are presented in Supplemental Figure 5 and Supplemental Figure 6, respectively. Mortality rates were very similar between California and the synthetic California from 2008 to 2010.

The corresponding plots of the gaps between each control state’s mortality rate and that of their corresponding synthetic control are presented for all control states (Supplemental Figure 7) and those within 5x (Supplemental Figure 8) and 2x (Supplemental Figure 9) of California’s pre-policy MSPE. Twenty-two (78.6%) of 28 control states (a solution did not converge for one state), 16 (76.2%) of 21 states within 5x of California’s pre-policy MSPE, and 10 (76.9%) of 13 states within 2x of California’s pre-policy MSPE had a post-policy MSPE greater than that of California. The plot of the ratios of post- to pre- policy MSPE for California and all control states is presented in Supplemental Figure 10. Nineteen (67.9%) of 28 control states had ratios of post- to pre-policy MSPE greater than that of California.

The effect size and corresponding permutation test when treating the implementation year as 2007 provide no evidence for an effect on California state prisoner mortality during this placebo period. These results demonstrate that the synthetic control method applied to these data was able to accurately reconstruct California’s observed mortality rates in the absence of a major intervention, increasing confidence in our estimates of the effects of AB109.

The leave-one-out estimates obtained when excluding single control states from the donor pool are presented in the Supplemental Figure 11. Exclusion of any of the states that contributed to the synthetic control unit in the main analysis had little impact on the estimates of AB109’s effect on mortality.

**Supplemental Tables and Figures**

| **Supplemental Table 1:** Overview of analyses and contributed data sources | | |
| --- | --- | --- |
| **Analysis** | **Contributing Data Sources** | **Data Source Components** |
| Evaluating effects of AB109 on crude mortality using synthetic control method | BJS Data | Annual mortality rates among California and comparison state inmates |
| Comparing changes in inmate age distribution between California and comparison states | NCRP Data | Age-specific annual incarcerated person-time among California and comparison state inmates, calculated from individual-level term records |
| Comparing trends in crude and age-standardized mortality rates among California inmates | DIC Data | Total and age-specific annual mortality counts among California inmates |
|  | NCRP Data | Age-specific annual incarcerated person-time among California inmates, calculated from individual-level term records |
| Comparing changes in age-standardized mortality rates between California and other states | BJS Data | Total annual mortality counts among comparison state inmates |
|  | NCRP Data | Age-specific annual incarcerated person-time among California and comparison state inmates, calculated from individual-level term records |
|  | DIC Data | Age-specific annual mortality counts among California inmates |
| BJS = Bureau of Justice Statistics; NCRP = National Corrections Reporting Program; DIC = Deaths in Custody Reporting Program | | |

| **Supplemental Table 2:** States included in crude and age-adjusted mortality analyses. | | |
| --- | --- | --- |
| **State** | **Included in Crude Mortality Analysis** | **Included in Age-Adjusted Mortality Analysis** |
| Alabama | X | X |
| Alaska^a,b,d^ |  |  |
| Arizona^d^ | X |  |
| Arkansas^d^ | X |  |
| California | X | X |
| Colorado^d^ | X |  |
| Connecticut^b,d^ |  |  |
| Delaware^a,b,d^ |  |  |
| Florida^c,d^ |  |  |
| Georgia^d^ | X |  |
| Hawaii^a,d^ |  |  |
| Idaho^a,d^ |  |  |
| Illinois | X | X |
| Indiana^d^ | X |  |
| Iowa^a,d^ |  |  |
| Kansas^d^ | X |  |
| Kentucky^d^ | X |  |
| Louisiana^d^ | X |  |
| Maine^a,d^ |  |  |
| Maryland | X | X |
| Massachusetts^d^ | X |  |
| Michigan | X | X |
| Minnesota^a,d^ |  |  |
| Mississippi^d^ | X |  |
| Missouri | X | X |
| Montana^a,d^ |  |  |
| Nebraska^a^ |  |  |
| Nevada | X | X |
| New Hampshire^a,d^ |  |  |
| New Jersey^d^ | X |  |
| New Mexico^a,d^ |  |  |
| New York | X | X |
| North Carolina | X | X |
| North Dakota^a,d^ |  |  |
| Ohio^d^ | X |  |
| Oklahoma^d^ | X |  |
| Oregon | X | X |
| Pennsylvania | X | X |
| Rhode Island^a,b^ |  |  |
| South Carolina | X | X |
| South Dakota^a,d^ |  |  |
| Tennessee^d^ | X |  |
| Texas^d^ | X |  |
| Utah^a,d^ |  |  |
| Vermont^a,b,d^ |  |  |
| Virginia^d^ | X |  |
| Washington | X | X |
| West Viginia^a,d^ |  |  |
| Wisconsin | X | X |
| Wyoming^a,d^ |  |  |
| ^a^Excluded from both crude and age-adjusted mortality analyses due to annual number of deaths < 10 during the study period. | | |
| ^b^Excluded from both crude and age-adjusted mortality analyses because prisons and jails form one integrated system in these states and mortality data were only available among the combined prison and jail populations. | | |
| ^c^Excluded from both crude and age-adjusted mortality analyses because the state privatized its prison healthcare system in 2011, which was reported to result in decreased quality of care. | | |
| ^d^Excluded from age-adjusted mortality analysis because yearend custody estimates calculated with NCRP data differed by more than 10% from those reported by BJS for at least one year during the period 2007-2014. | | |

| **Supplemental Table 3:** Differences between annual number of deaths among California state prisoners included in Deaths in Custody (DIC) data and reported by the Bureau of Justice Statistics (BJS). | | | |
| --- | --- | --- | --- |
| **Year** | **Included in Deaths in Custody (DIC) Data** | **Reported by The Bureau of Justice Statistics (BJS)** | **Relative Difference*** |
| 2008 | 355 | 371 | 4.3% |
| 2009 | 392 | 395 | 0.8% |
| 2010 | 413 | 414 | 0.2% |
| 2011 | 386 | 388 | 0.5% |
| 2012 | 363 | 368 | 1.4% |
| 2013 | 360 | 366 | 1.6% |
| 2014 | 313 | 317 | 1.3% |
| *Calculated with the BJS counts as the reference. | | | |

| **Supplemental Table 4:** Annual number of deaths among California medical parolees, 2011-2014 | |
| --- | --- |
| **Year** | **Count of Medical Parole Deaths** |
| 2011 | 5 |
| 2012 | 7 |
| 2013 | 7 |
| 2014 | 4 |

| **Supplemental Table 5:** Composition of Synthetic Control | |
| --- | --- |
| **Control State** | **Weight** |
| Illinois | 0.301 |
| Missouri | 0.229 |
| Oregon | 0.166 |
| Washington | 0.154 |
| Indiana | 0.079 |
| Arizona | 0.044 |
| Georgia | 0.027 |

| **Supplemental Table 6:** Age-specific mortality counts among California state prisoners, 2008-2014* | | | | | | | |
| --- | --- | --- | --- | --- | --- | --- | --- |
| **Age Group** | **2008** | **2009** | **2010** | **2011** | **2012** | **2013** | **2014** |
| **Under 25** | <10^†^ | <10^†^ | <10^†^ | <10^†^ | <10^†^ | <10^†^ | <10^†^ |
| **25-34** | 28 | 26 | 37 | 25 | 21 | 25 | 22 |
| **35-44** | 55 | 42 | 57 | 46 | 43 | 42 | 27 |
| **45-54** | 118 | 129 | 110 | 106 | 98 | 86 | 81 |
| **55-64** | 81 | 110 | 122 | 117 | 104 | 104 | 84 |
| **65-74** | 48 | 50 | 58 | 50 | 60 | 67 | 65 |
| **Over 74** | 21 | 28 | 25 | 33 | 31 | 29 | 28 |
| *Death counts from Deaths in Custody (DIC) data | | | | | | | |
| ^†^Counts <10 are suppressed | | | | | | | |

| **Supplemental Table 7:** Age-specific mortality rates among California state prisoners, 2008-2014* | | | | | | | |
| --- | --- | --- | --- | --- | --- | --- | --- |
| **Age Group** | **2008** | **2009** | **2010** | **2011** | **2012** | **2013** | **2014** |
| **Under 25** | 17.2 | 30.5 | 17.7 | 41.7 | 32.9 | 40.1 | 35.3 |
| **25-34** | 49.7 | 47.7 | 69.7 | 49.3 | 49.8 | 61.0 | 52.2 |
| **35-44** | 113.1 | 91.1 | 129.7 | 111.0 | 122.0 | 123.6 | 78.4 |
| **45-54** | 352.7 | 381.0 | 324.2 | 318.4 | 335.0 | 309.0 | 295.6 |
| **55-64** | 895.4 | 1113.4 | 1140.0 | 1024.9 | 913.1 | 883.0 | 680.0 |
| **65-74** | 2605.6 | 2483.4 | 2590.5 | 2062.6 | 2299.8 | 2389.1 | 2136.7 |
| **Over 74** | 5576.0 | 6938.3 | 5620.2 | 6633.2 | 5925.2 | 5192.8 | 4714.5 |
| *Rates calculated using death counts from Deaths in Custody (DIC) data and incarcerated person-time from National Corrections Reporting Program (NCRP) data. | | | | | | | |

| **Supplemental Table 8:** Age-adjusted excess mortality rates before and after the implementation of AB109, 13 states | | | | | |
| --- | --- | --- | --- | --- | --- |
|  | **Pre-Policy Period** |  | **Post-Policy Period** | | |
| **State** | **2010*** |  | **2012†** | **2013†** | **2014†** |
| Alabama | 72.4 |  | -72.8 | -141.0 | -156.8 |
| Illinois | -11.4 |  | 22.7 | 43.5 | -3.9 |
| Maryland | 149.6 |  | -27.0 | -77.2 | -27.5 |
| Michigan | 20.2 |  | -82.9 | -78.3 | -99.2 |
| Missouri | 27.1 |  | 57.2 | -4.9 | -49.0 |
| North Carolina | 108.6 |  | 14.6 | 14.1 | -48.0 |
| Nevada | -16.8 |  | 37.3 | 58.8 | -46.2 |
| New York | 32.9 |  | 4.0 | -48.3 | -48.0 |
| Oregon | -3.2 |  | 77.7 | 54.0 | 12.8 |
| Pennsylvania | 68.3 |  | -35.5 | -30.5 | -39.5 |
| South Carolina | 84.4 |  | 35.8 | 5.5 | -29.3 |
| Washington | 36.4 |  | 7.9 | -17.4 | -47.1 |
| Wisconsin | 115.3 |  | -93.6 | -69.9 | -165.3 |
| **MEDIAN** | **36.4** |  | **7.9** | **-17.4** | **-47.1** |
| *Refers to change in mortality from 2008 to 2010. | | | | | |
| †Refers to change in mortality from 2010 to each post-policy year. | | | | | |

| **Supplemental Table 9:** 2.5% and 97.5% quantiles of median age-adjusted excess mortality rates over 1000 iterations of stochastically correcting DIC death counts to match those reported by BJS, 13 states | | |
| --- | --- | --- |
| **Post-Policy Year*** | **2.5% Quantile** | **97.5% Quantile** |
| 2012 | 10.5 | 11.3 |
| 2013 | -14.1 | -13.2 |
| 2014 | -45.2 | -44.6 |
| **Pre-Policy Year†** | **2.5% Quantile** | **97.5% Quantile** |
| 2010 | 26.3 | 27.6 |
| *Refers to change in mortality from 2010 to each post-policy year. | | |
| †Refers to change in mortality from 2008 to 2010. | | |

| **Supplemental Table 10:** Median age-adjusted excess mortality rate and exact confidence intervals calculated with the inclusion of California medical parole deaths**,** 13 states | | |
| --- | --- | --- |
| **Post-Policy Year*** | **Median** | **(97.8% CI)** |
| 2012 | 12.4 | (-68.6, 42.3) |
| 2013 | -12.3 | (-73.8, 46.4) |
| 2014 | -44.3 | (-96.3, -25.1) |
| *Refers to change in mortality from 2010 to each post-policy year. | | |

| **Supplemental Table 11:**  2.5% and 97.5% quantiles of median age-adjusted excess mortality rates over 1000 iterations of stochastically correcting DIC death counts to match those reported by BJS and adding California medical parole deaths, 13 states | | |
| --- | --- | --- |
| **Post-Policy Year*** | **2.5% Quantile** | **97.5% Quantile** |
| 2012 | 15.0 | 15.7 |
| 2013 | -8.9 | -8.0 |
| 2014 | -42.4 | -41.8 |
| *Refers to change in mortality from 2010 to each post-policy year. | | |

**Supplemental Figure 1**


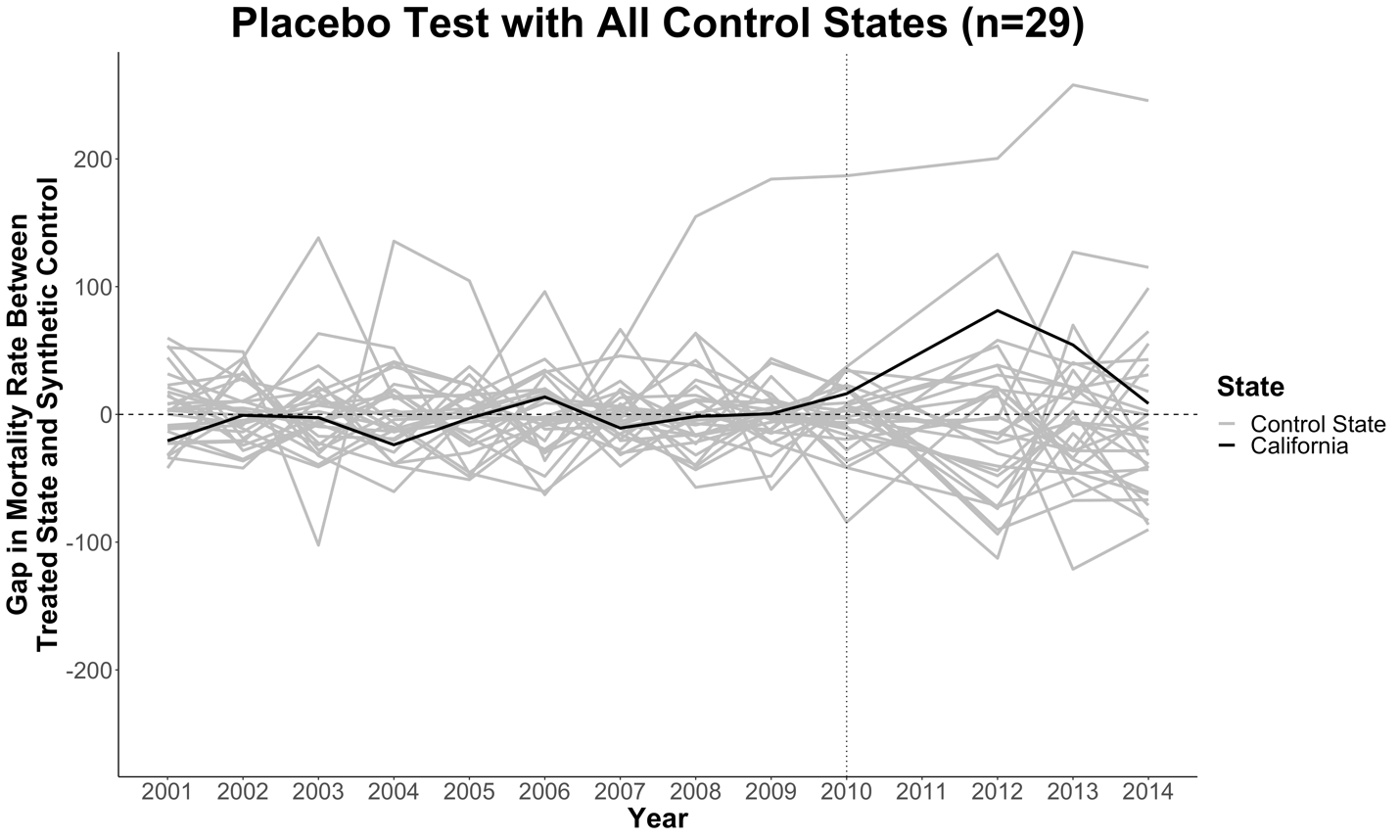


**Supplemental Figure 2**


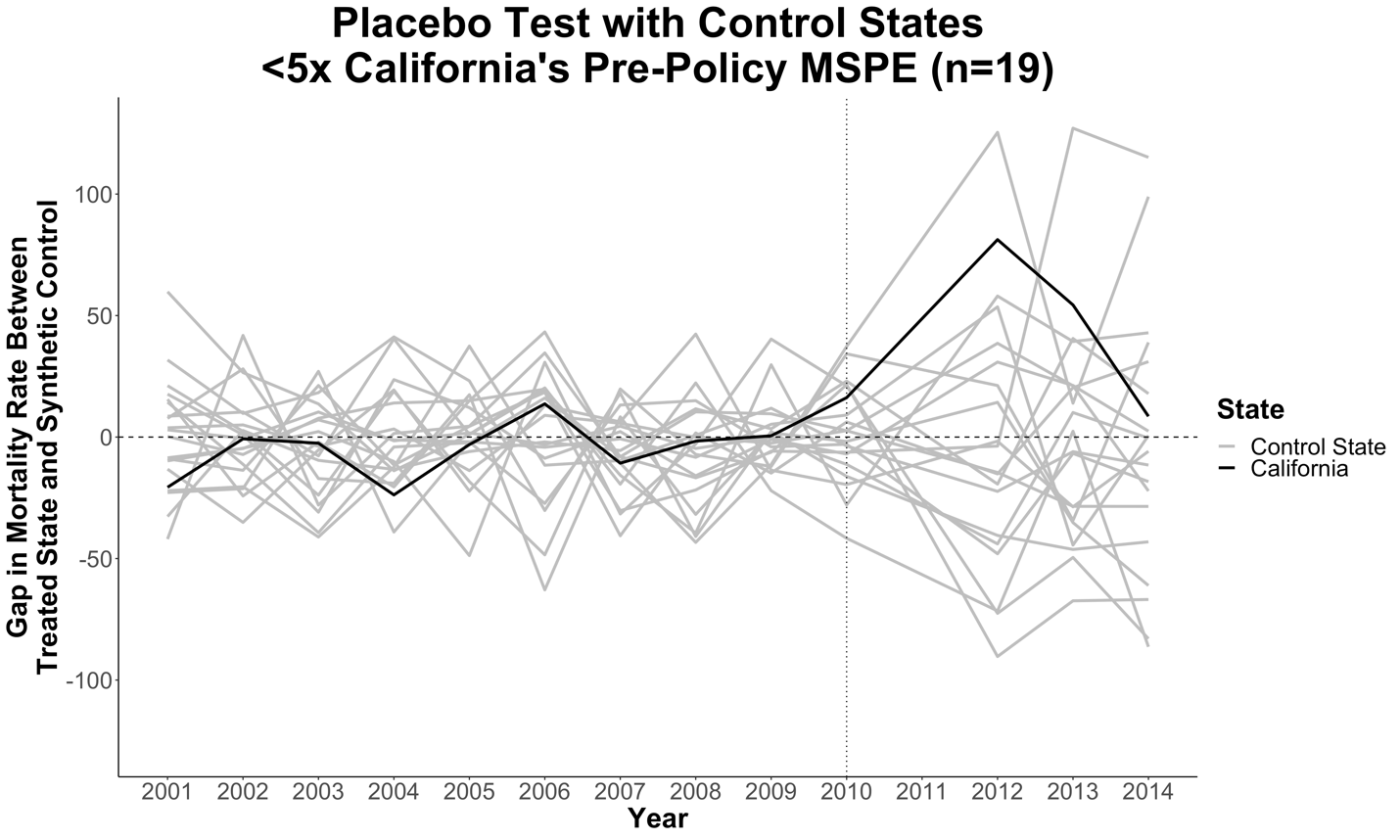


**Supplemental Figure 3**


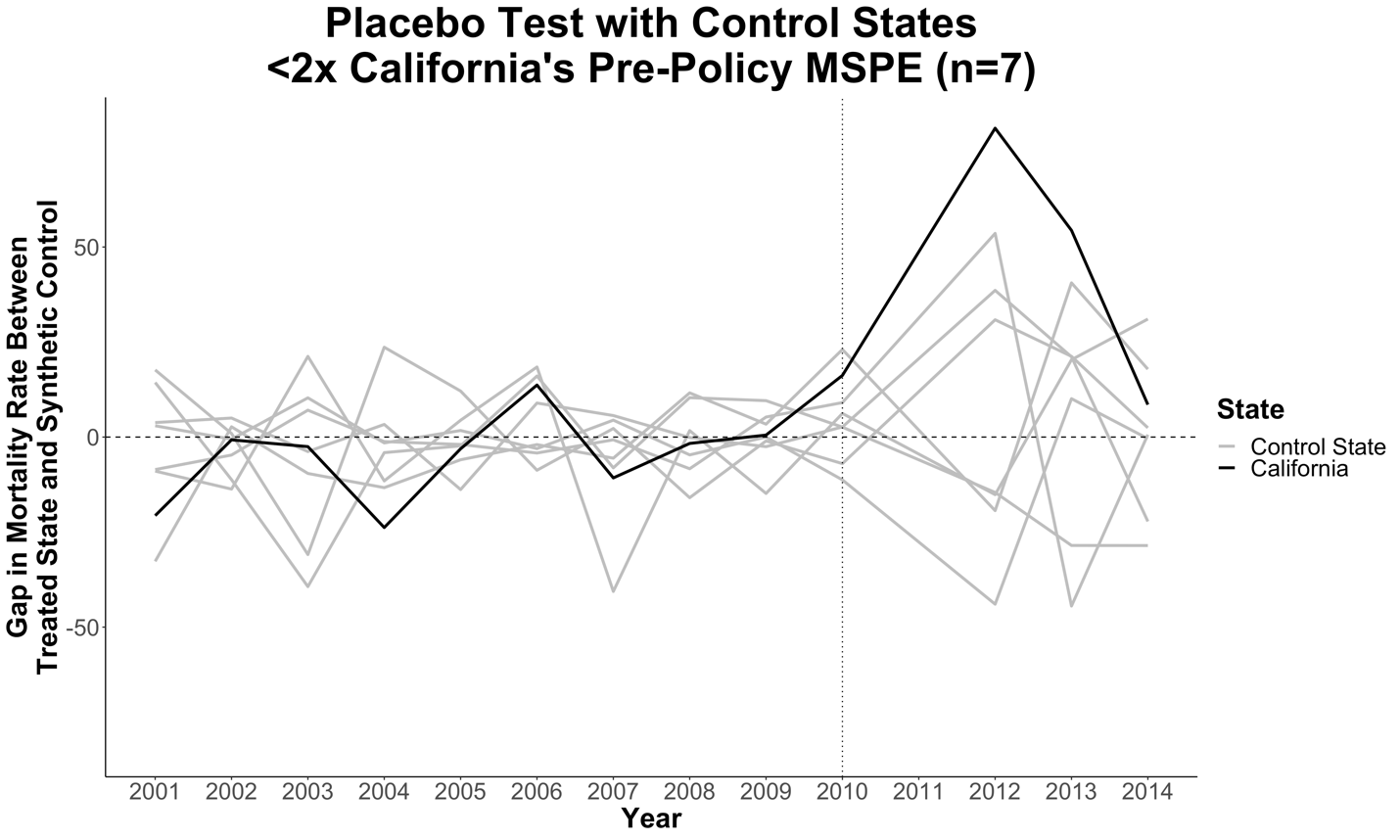


**Supplemental Figure 4**


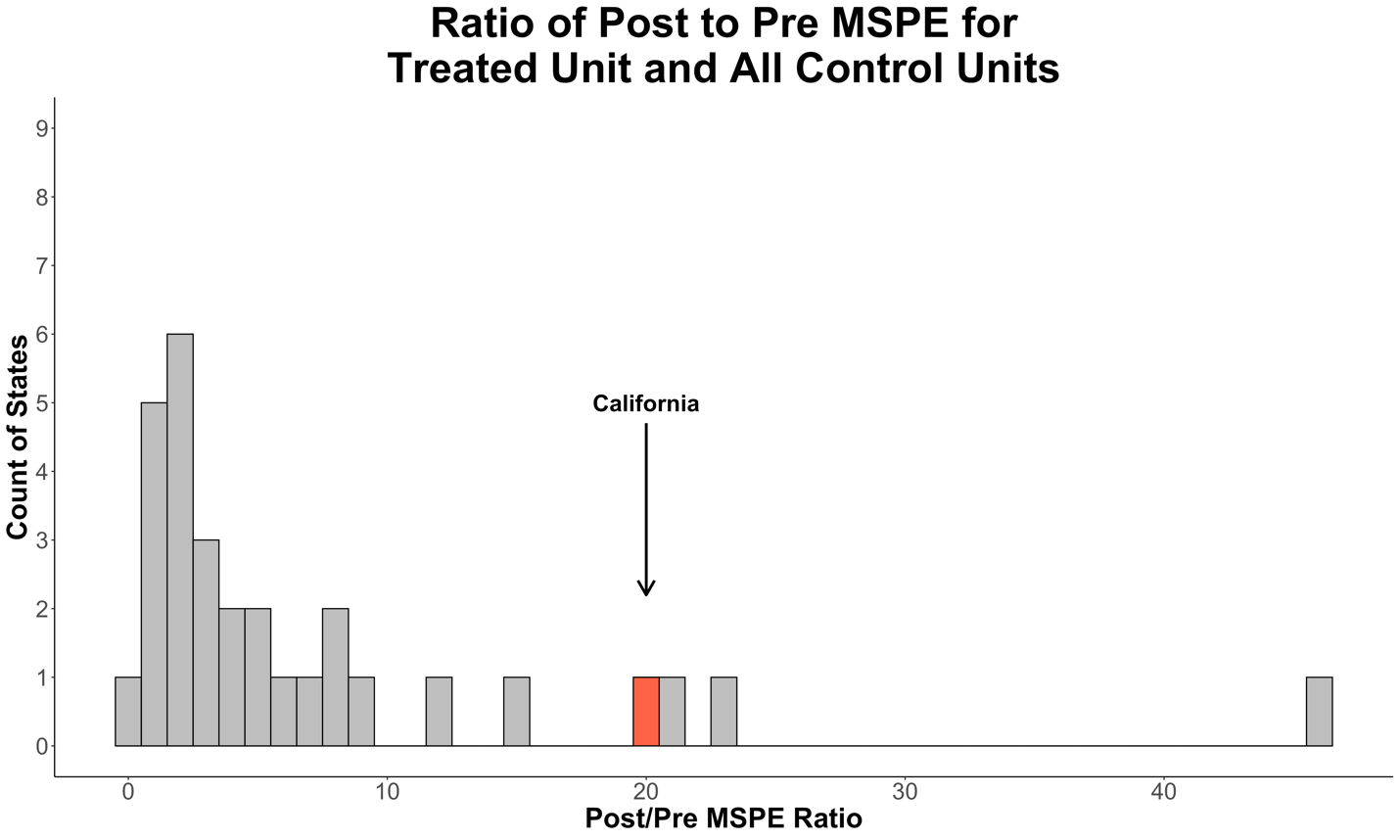


**Supplemental Figure 5**


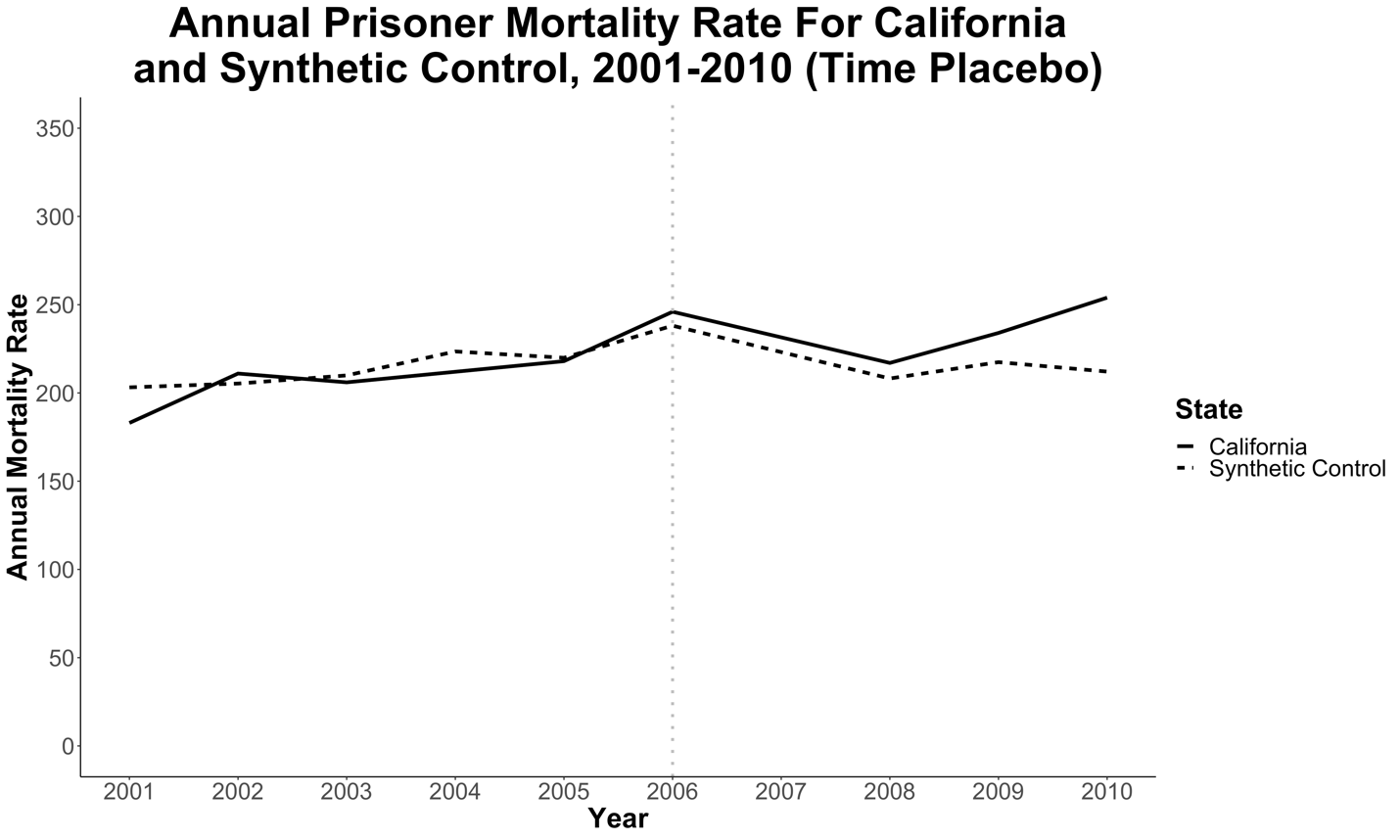


**Supplemental Figure 6**


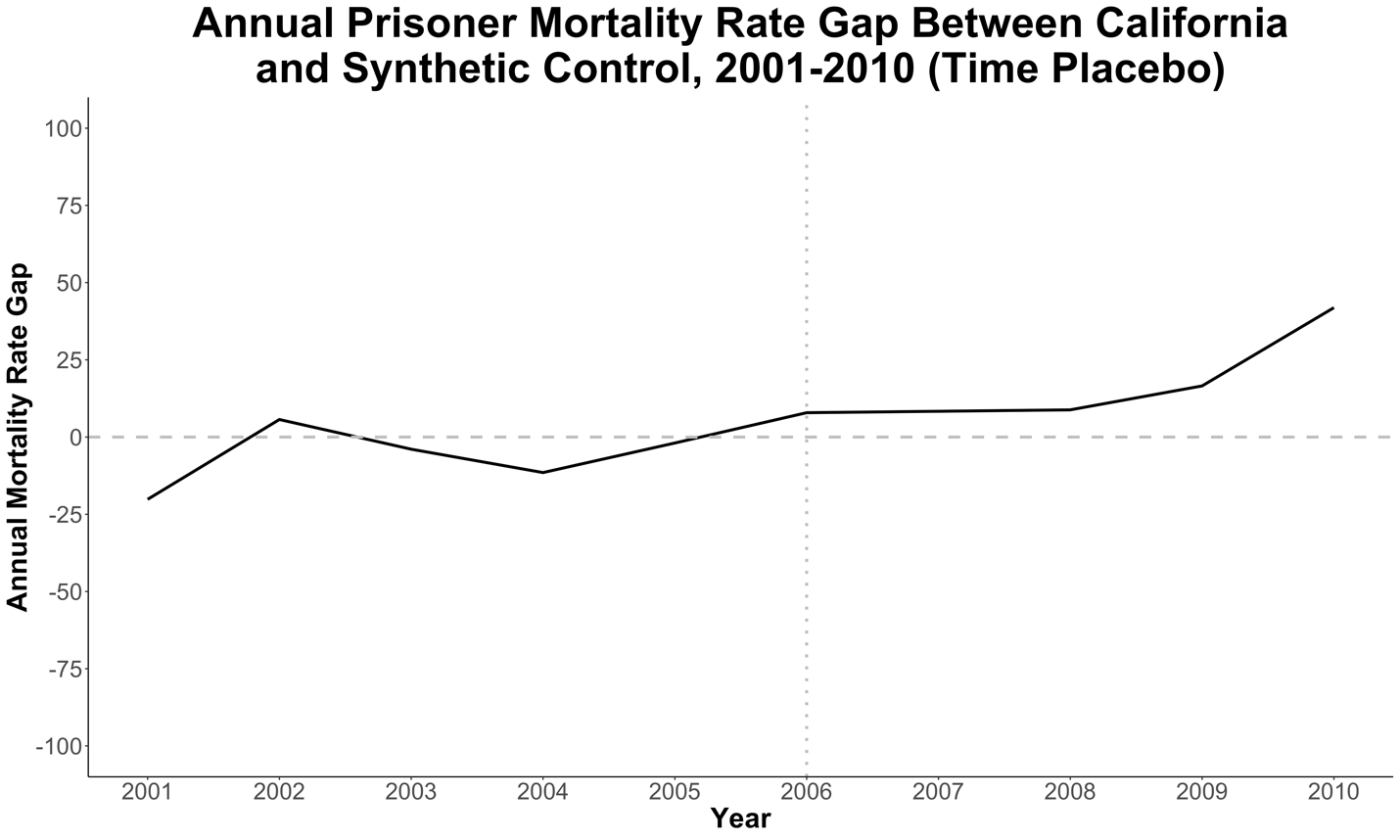


**Supplemental Figure 7**


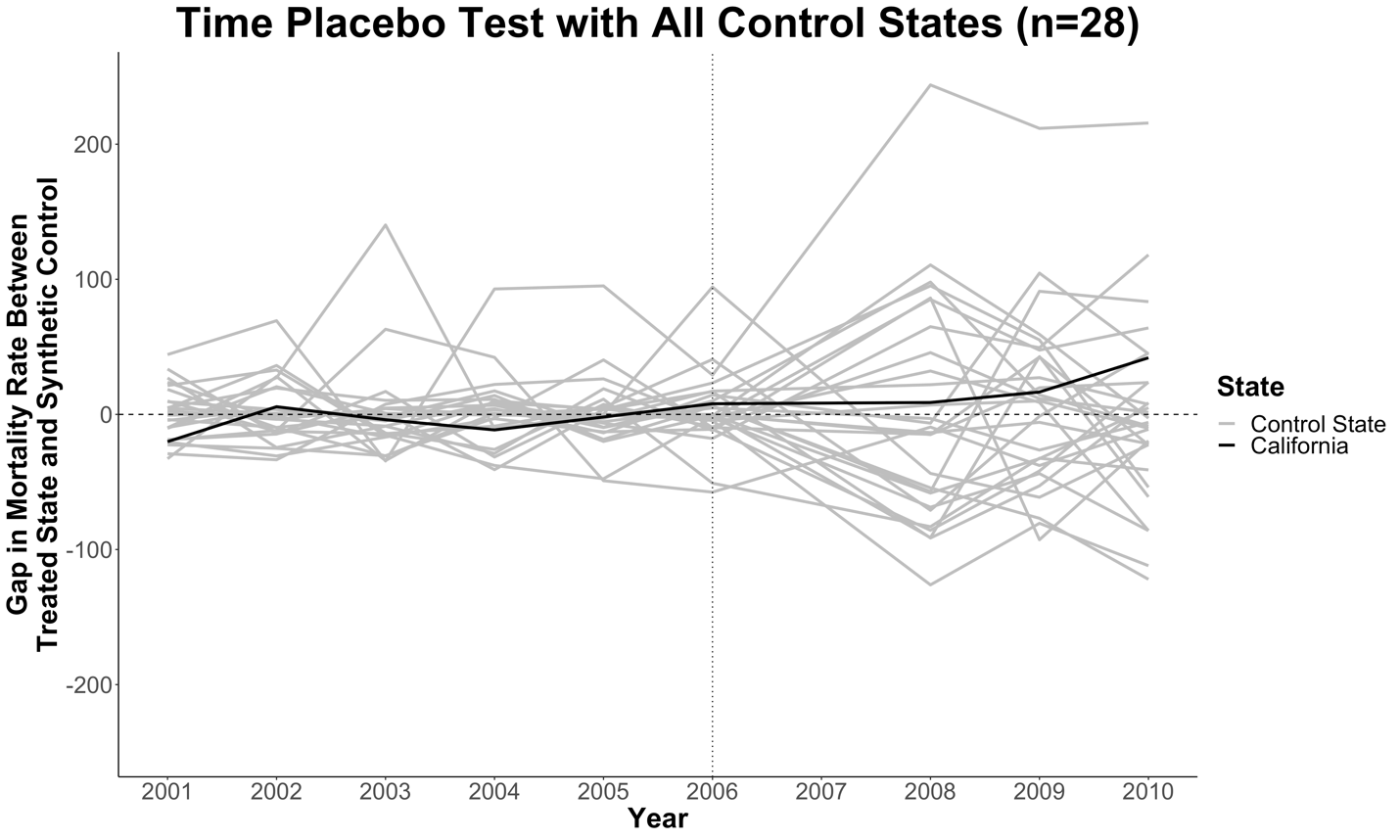


**Supplemental Figure 8**


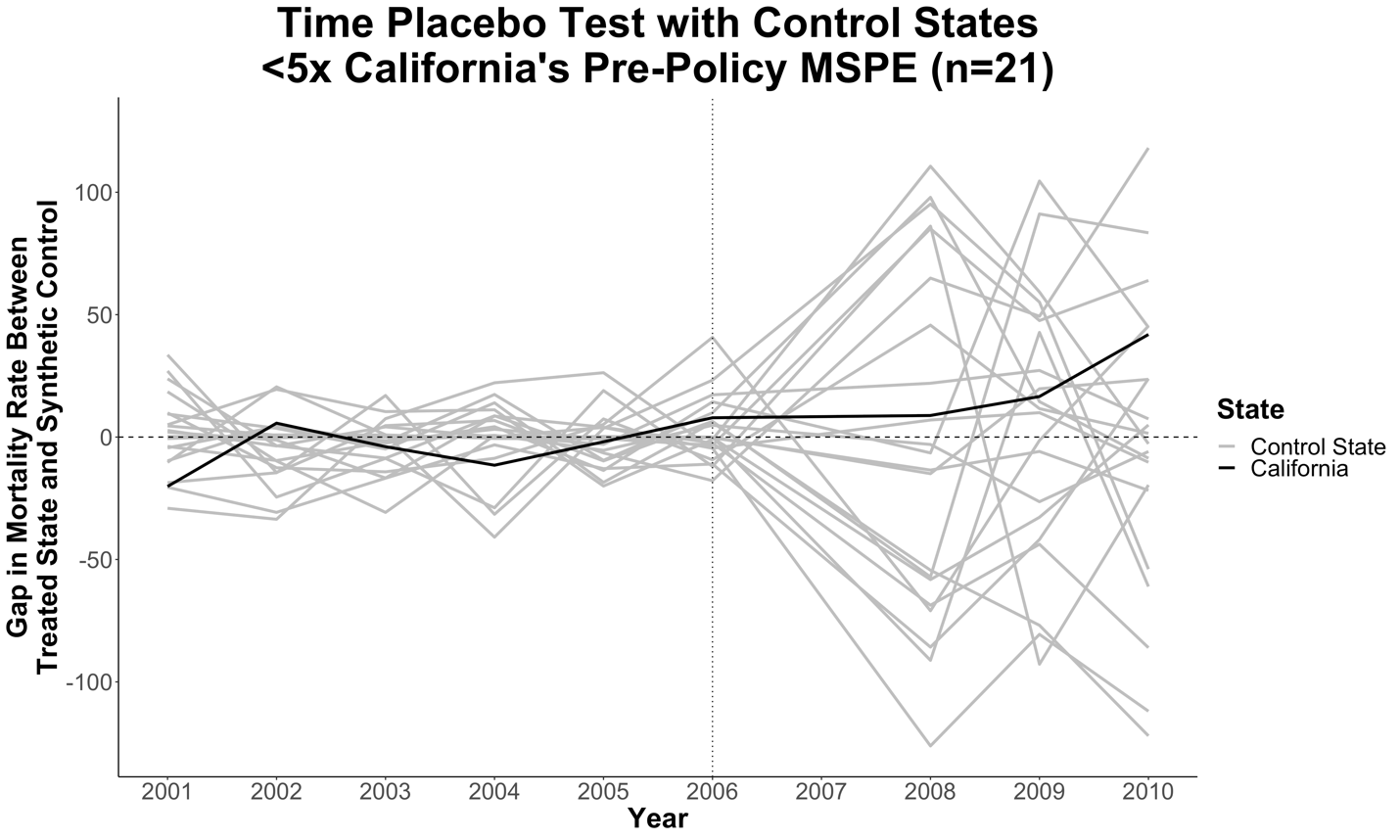


**Supplemental Figure 9**


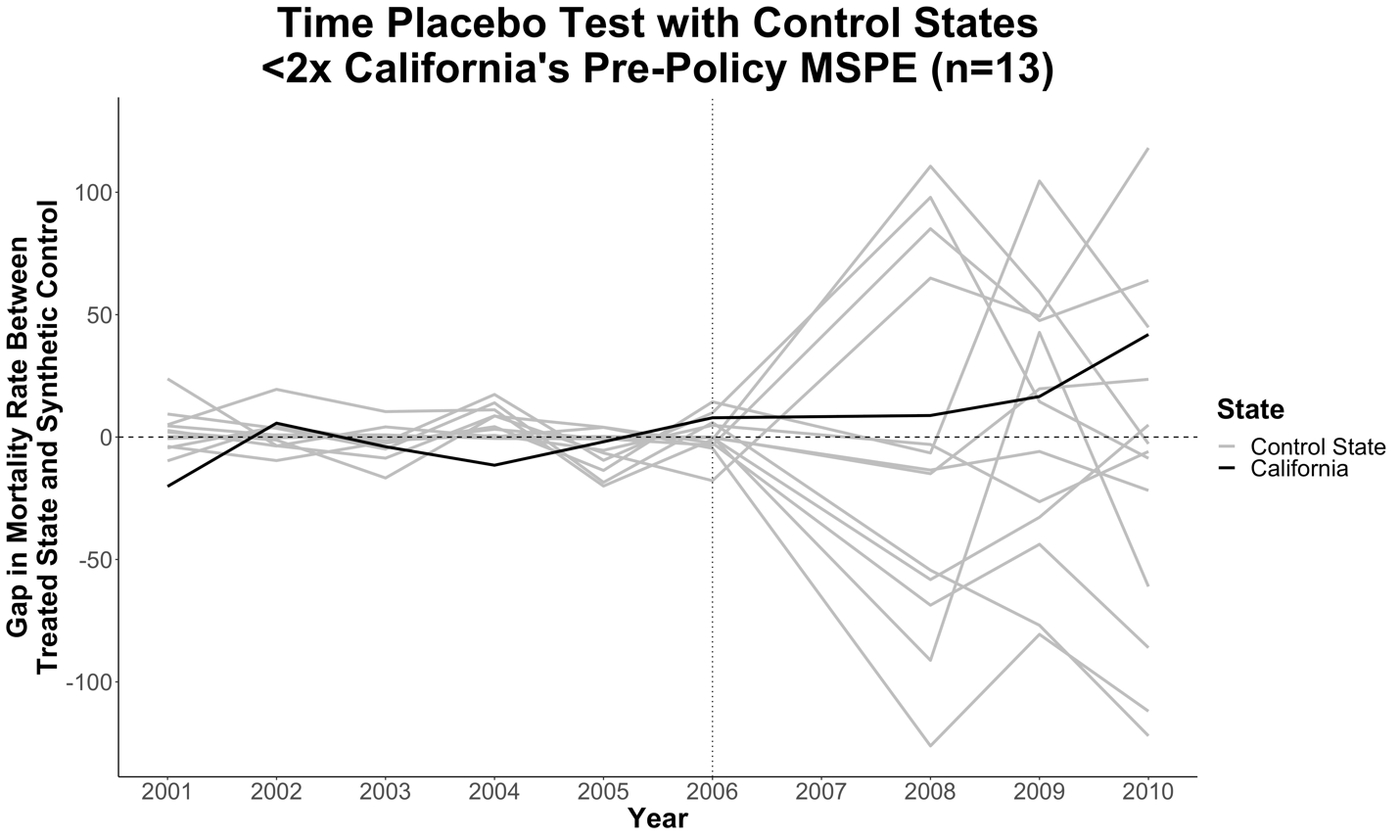


**Supplemental Figure 10**


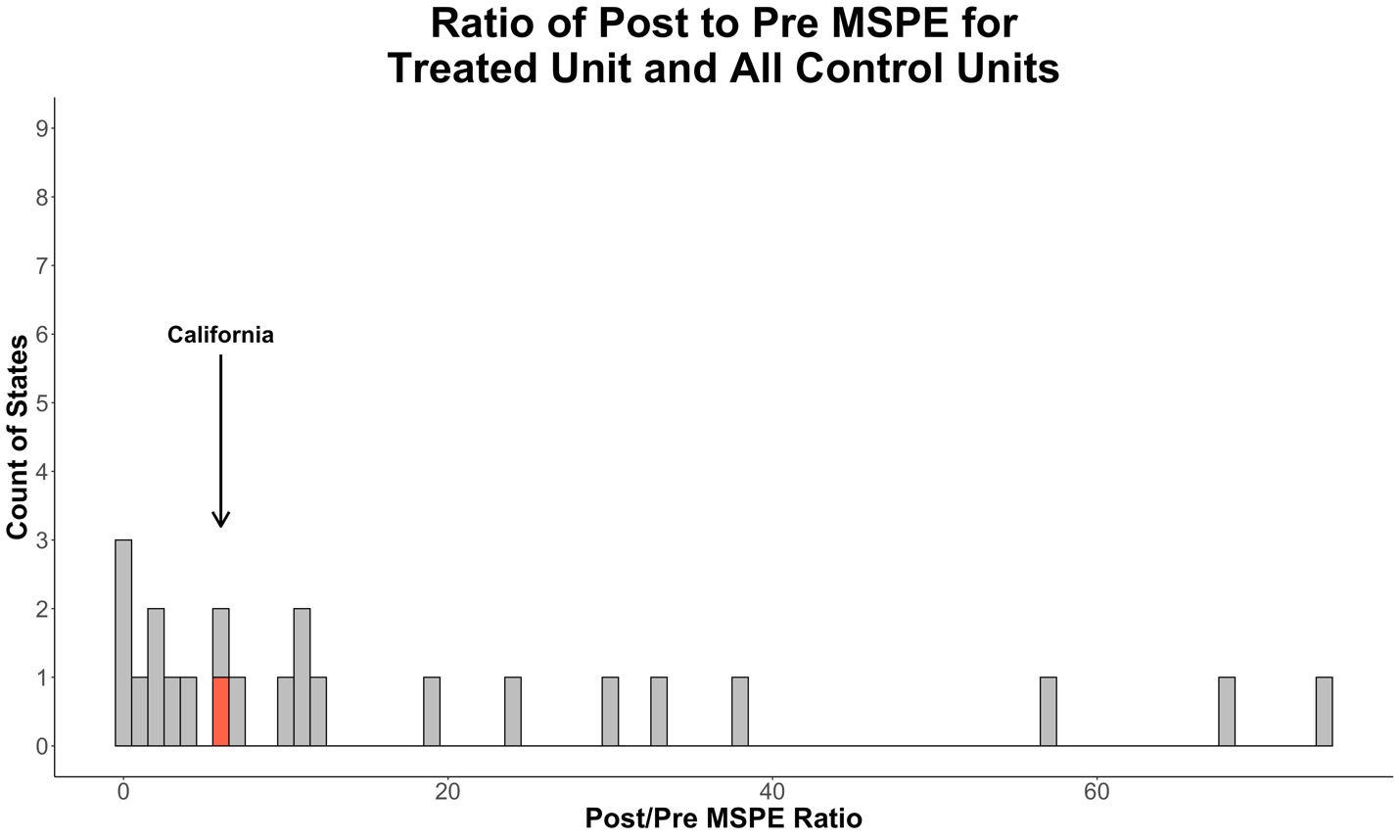


**Supplemental Figure 11**


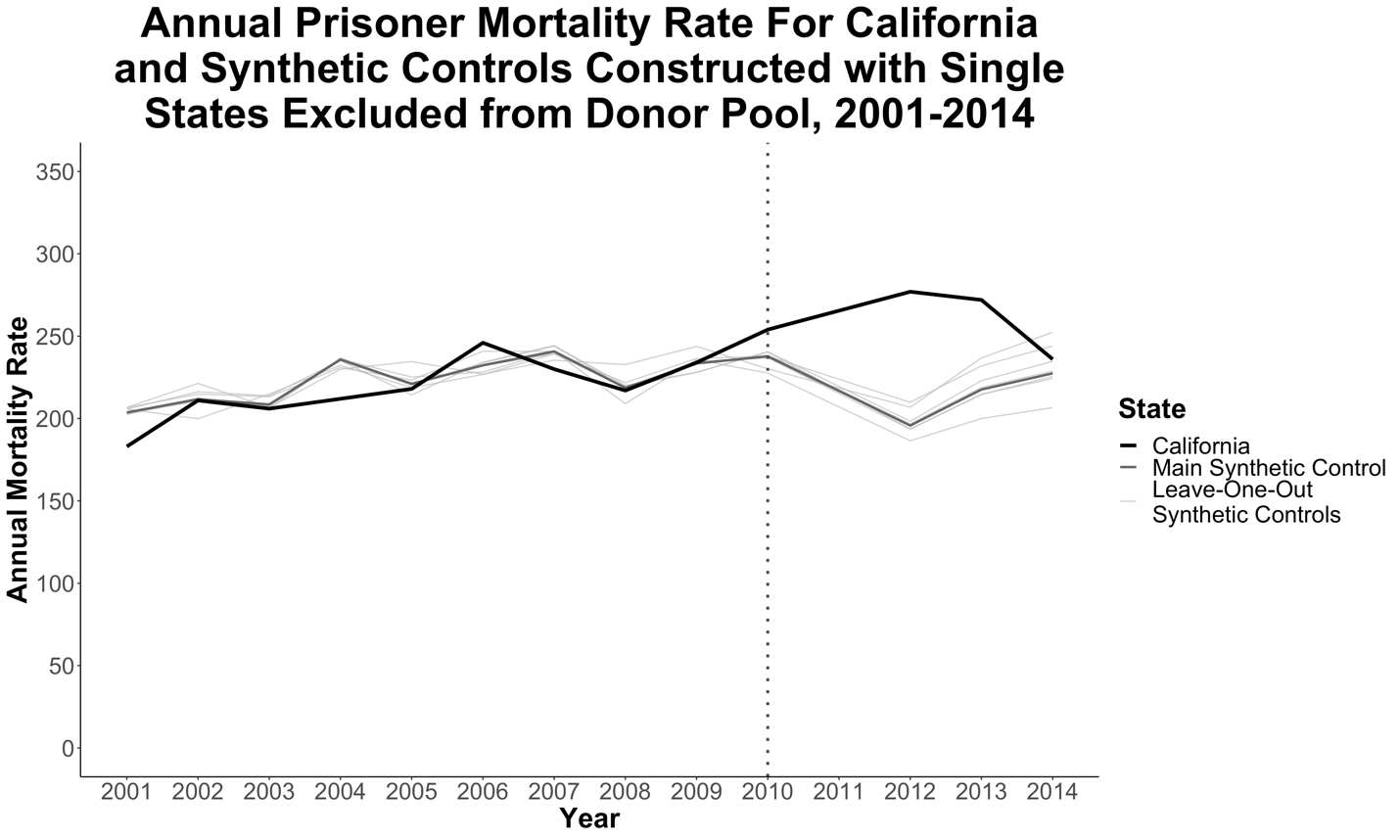


**Supplemental Figure 12**


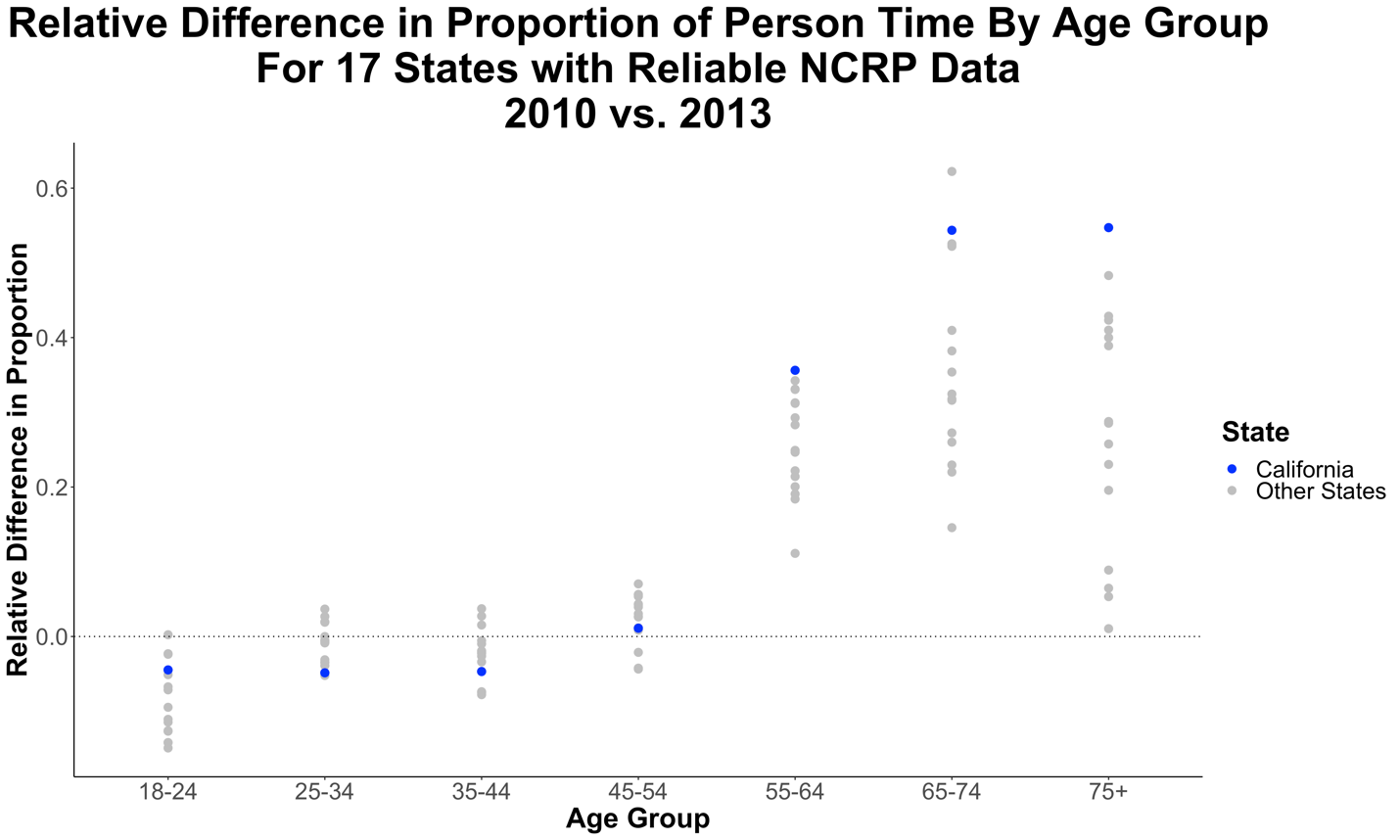


**Supplemental Figure 13**

**
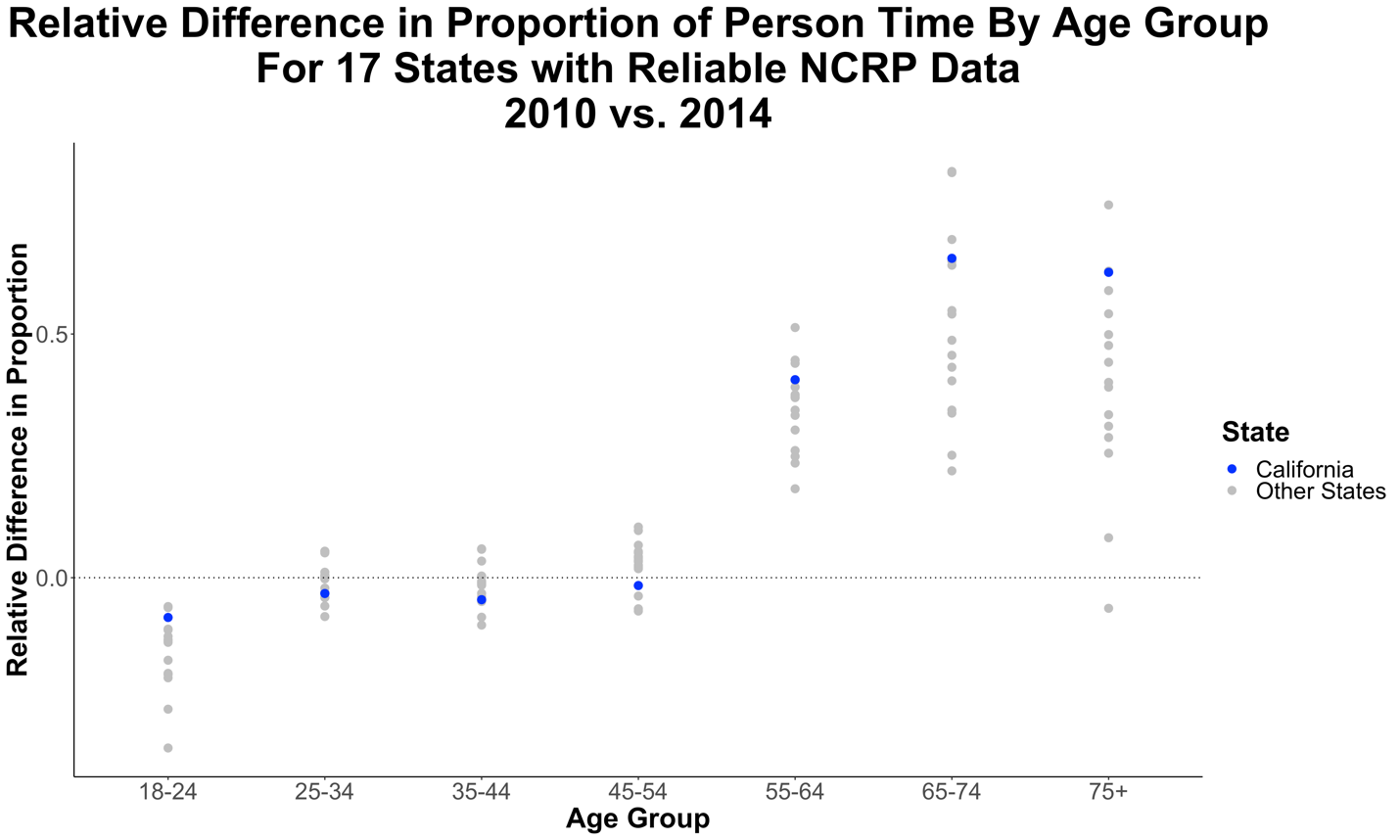
**

**Supplemental Figure 14**

**
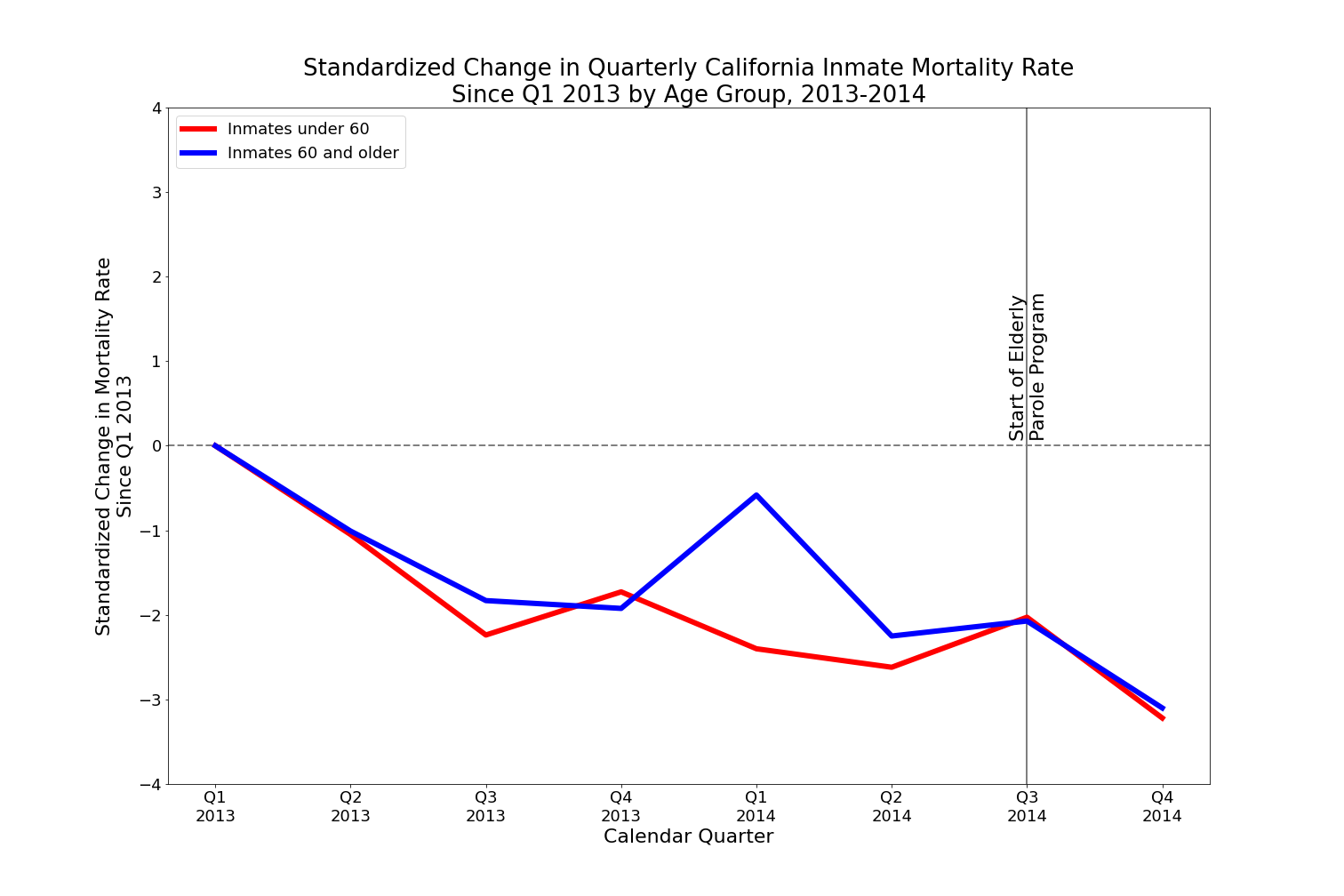
**

**REFERENCES**

1. Centers for Disease Control and Prevention National Center for Health Statistics. Multiple Cause of Death 1999-2018 on CDC WONDER Online Database, released 2019. March 2018. http://wonder.cdc.gov/.

2. United States Department Of Justice. Office Of Justice Programs. Bureau Of Justice Statistics. Prisoners Series. https://www.bjs.gov/index.cfm?ty=pbse&sid=40. Accessed November 30, 2020.

3. Prison Policy Initiative. Percentage of incarcerated state prison population 55 years of age and older for each state, with comparative total prison population counts. https://www.prisonpolicy.org/data/older_prisonpopulations.xlsx. Accessed November 30, 2020.

4. Abadie A, Diamond A, Hainmueller J. Synthetic control methods for comparative case studies: Estimating the effect of California’s tobacco control program. *Journal of the American Statistical Association*. 2010;105(490):493-505.

5. Abadie A, Diamond A, Hainmueller J. Comparative Politics and the Synthetic Control Method. *American Journal of Political Science*. 2015;59(2):495-510. doi:10.1111/ajps.12116

6. Carson EA, Cowhig MP. *Mortality in State and Federal Prisons, 2001-2016 - Statistical Tables*. United States Department of Justice, Office of Justice Programs, Bureau of Justice Statistics; 2020.

7. Noonan ME. *Mortality in State Prisons, 2001-2014 - Statistical Tables*. United States Department of Justice, Office of Justice Programs, Bureau of Justice Statistics; 2016.

8. Carter Goble Associates, LLC. *Health Care Study: Florida Department of Corrections*.; 2019. https://oppaga.fl.gov/Products/ReportDetail?rn=19-FDCMed. Accessed August 18, 2020.

9. Carson EA, Mulako-Wangota J. Count of custody population - sentences less than or equal to 1 year. Generated using the Corrections Statistical Analysis Tool (CSAT)-Prisoners at www.bjs.gov.

10. Carson EA, Mulako-Wangota J. Count of custody population - sentences greater than 1 year. Generated using the Corrections Statistical Analysis Tool (CSAT)-Prisoners at www.bjs.gov.

11. Neal D, Armin R. The Prison Boom and the Lack of Black Progress after Smith and Welch. *NBER Working Paper No w20283*. July 2014.

12. Pfaff JF. The Myths and Realities of Correctional Severity: Evidence from the National Corrections Reporting Program on Sentencing Practices. *American Law and Economics Review*. 2011;13(2):491-531. doi:10.1093/aler/ahr010
